# Rational Design of Three Dimensional Hollow Heterojunctions for Efficient Photocatalytic Hydrogen Evolution Applications

**DOI:** 10.1002/advs.202309293

**Published:** 2024-01-23

**Authors:** Jingwen Pan, Dongbo Wang, Donghai Wu, Jiamu Cao, Xuan Fang, Chenchen Zhao, Zhi Zeng, Bingke Zhang, Donghao Liu, Sihang Liu, Gang Liu, Shujie Jiao, Zhikun Xu, Liancheng Zhao, Jinzhong Wang

**Affiliations:** ^1^ School of Materials Science and Engineering Harbin Institute of Technology Harbin 150001 China; ^2^ Henan Key Laboratory of Nanocomposites and Applications Huanghe Science and Technology College Institute of Nanostructured Functional Materials Zhengzhou 450006 China; ^3^ School of Astronautics Harbin Institute of Technology Harbin 150001 China; ^4^ State Key Lab High Power Semicond Lasers Changchun University Science and Technology, Sch Sci Changchun 130022 China; ^5^ Center for High Pressure Science and Technology Advanced Research Shanghai 201203 China; ^6^ Guangdong University of Petrochemical Technology Maoming 525000 China

**Keywords:** 3D hollow heterojunctions, built‐in electric field, Cu_2_O─S@GO@Zn_0.67_Cd_0.33_S, photocatalysis, photothermal effect

## Abstract

The efficiency of photocatalytic hydrogen evolution is currently limited by poor light adsorption, rapid recombination of photogenerated carriers, and ineffective surface reaction rate. Although heterojunctions with innovative morphologies and structures can strengthen built‐in electric fields and maximize the separation of photogenerated charges. However, how to rational design of novel multidimensional structures to simultaneously improve the above three bottleneck problems is still a research imperative. Herein, a unique Cu_2_O─S@graphene oxide (GO)@Zn_0.67_Cd_0.33_S Three dimensional (3D) hollow heterostructure is designed and synthesized, which greatly extends the carrier lifetime and effectively promotes the separation of photogenerated charges. The H_2_ production rate reached 48.5 mmol g^−1^ h^−1^ under visible light after loading Ni^2+^ on the heterojunction surface, which is 97 times higher than that of pure Zn_0.67_Cd_0.33_S nanospheres. Furthermore, the H_2_ production rate can reach 77.3 mmol g^−1^ h^−1^ without cooling, verifying the effectiveness of the photothermal effect. Meanwhile, in situ characterization and density flooding theory calculations reveal the efficient charge transfer at the p‐n 3D hollow heterojunction interface. This study not only reveals the detailed mechanism of photocatalytic hydrogen evolution in depth but also rationalizes the construction of superior 3D hollow heterojunctions, thus providing a universal strategy for the materials‐by‐design of high‐performance heterojunctions.

## Graphical abstract

Herein, a unique Cu_2_O─S@graphene oxide (GO)@Zn_0.67_Cd_0.33_S 3D hollow heterostructure is designed to realize the spatial separation and effective transfer of photogenerated charges. This study not only reveals the detailed mechanism of photocatalytic hydrogen evolution in depth through in situ characterization and theoretical calculations but also provides a universal strategy for the rational construction of excellent hollow heterojunctions.

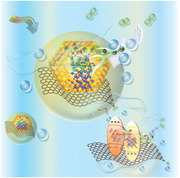

## 1 Introduction

The global energy crisis and environmental pollution caused by the excessive consumption of fossil energy sources have caused widespread concern worldwide.^[^ ^1^, ^2^ ^]^ Considering solar energy as one of the most abundant and green renewable energy sources, solar‐chemical energy conversion/storage by driving the production of solar fuels or accelerating chemical conversion is one of the most promising alternatives to address the above global challenges. Photocatalytic mimicry of photosynthesis has been designed to generate solar fuels such as hydrogen through the process of water splitting, which is expected to lead to a sustainable supply of carbon‐neutral clean energy.^[^ ^3^, ^4^ ^]^ Hydrogen (H_2_) is an environmentally friendly energy source with high energy density and no secondary pollution.^[^ ^5^ ^]^ Photocatalysis, the direct conversion of sunlight to chemical energy, has been widely studied over the last few decades.^[^ ^6^, ^7^ ^]^ The efficiency of photocatalytic H_2_ production depends on three primary factors: 1) the light absorption efficiency, 2) the separation and transfer efficiency of photogenerated electron‐hole pairs, and 3) the surface/interface reaction efficiency.^[^ ^8^, ^9^ ^]^ After photoexcitation, the generated carriers can diffuse toward the surface of the photocatalyst; however, during this process, electrons and holes tend to recombine on the photocatalyst as a whole or on the surface (Step 2). This is one of the major losses in the efficiency of solar energy utilization, and improving charge separation has received extensive attention.^[^ ^10^ ^]^ To develop efficient photocatalytic systems, it is important to design effective mechanisms to control the kinetics of charge transport processes (e.g., photogenerated charge generation, separation, and transfer). Therefore, shortening the charge transfer distance by nanostructure engineering or creating a built‐in electric field through junctions are the two main strategies to reduce charge complexity.

Hollow nanostructures have been widely studied due to their unique properties: 1) hollow nanostructures are favorable for light trapping; 2) thin shell layers effectively reduce carrier transport distances and thus charge complexation; hollow structures provide high specific surface areas; and 4) shell layers facilitate the realization of spatial separation of redox reactions.^[^ ^11^, ^12^ ^]^ In particular, the hollow structure can reduce the resistance to the formation of compact structures and increase the connectivity between the active species and the reaction site, thereby improving the catalytic performance. In general, the design of hollow nano knots can be categorized into three methods based on the formwork: the hard formwork method, the soft formwork method, and the no‐formwork/self‐formwork method.^[^ ^13^, ^14^ ^]^ In previous studies, templates such as SiO_2_ nanospheres have been used for preparing hollow structures; their use requires additional dissolution steps and controlled conditions to obtain regular spherical shapes.^[^ ^15^, ^16^ ^]^ Compared to hard formwork, soft formwork has less control over the product, such as shape, shell thickness, and dimensional uniformity.^[^ ^17^ ^]^ Direct synthesis without additional templates is favorable for the consideration of industrial applications because of the significant reduction of production costs and scale‐up feasibility. Therefore, the construction of hollow nanostructures by template‐free/self‐templated methods has been widely investigated.^[^ ^18^ ^]^ Anisotropic nanocages with nonspherical shapes and core regions are of particular interest, owing to their unique hollow polyhedral structures. Based on their elemental abundance and low cost, Cu_2_O polyhedra are suitable templates for the design of 3D structures.^[^ ^19^, ^20^ ^]^ Cu_2_O nanocrystals with octahedral and more complex structures have been prepared by controlling the synthesis conditions, and are also easy to dissolve into hollow structures through various strategies, such as chemical etching or the Kirkendall effect. The introduction of an additional built‐in electric field is another important strategy while considering the advantages of hollow structures for photocatalysts in terms of charge separation and transport.

Typically, p‐n heterojunctions are the result of a large difference in the Fermi energy levels of the two, effectively creating a strong internal electric field that provides a significant electrostatic force that regulates charge transfer.^[^ ^21^, ^22^ ^]^ The construction of a heterojunction can create a situation where the interface between two semiconductors exhibits different electric potentials, and the potential difference leads to a charge redistribution to offset the different Fermi energy levels (E*_f_*). In general, electrons tend to cluster on the crystal face with the lowest E*_f_*, while holes stay on the crystal face with the highest E*_f_*.^[^ ^23^ ^]^ As a result, energy band bending in opposite directions occurs near the two faces, creating an internal electric field directed from the region with the higher E*_f_* to that with the lower E*_f_*.^[^ ^2^ ^]^ In the presence of an external driving force, both the charge separation and transfer efficiencies are increased, leading to an enhanced photocatalytic hydrogen production performance.^[^ ^24^, ^25^ ^]^ Zn_x_Cd_1−x_S solid solutions with tunable composition and energy band structure have typical n‐type structures and excellent light absorption properties.^[^ ^26^, ^27^ ^]^ Thus, these solid solutions are appropriate for the construction of novel 3D heterojunctions based on p‐Cu_2_O and n‐Zn_x_Cd_1−x_S. Heterojunction structures can overcome the light absorption and redox deficiencies of Cu_2_O and strengthen the built‐in electric field to enhance the efficiency of charge separation and interfacial charge transfer.

Effectively utilizing the abundant light and heat energy generated from solar energy, the development of photocatalysts with a photothermal coupling effect is another key solution to improve the performance of photocatalytic hydrogen evolution. To date, various carbon‐based materials such as graphene oxide (GO)/reduced graphene oxide (rGO) and carbon nanotubes have been explored.^[^ ^28^, ^29^ ^]^ Based on its 2D nature, superior electrical conductivity, and atomic‐layer thickness, graphene can increase the transfer rate of photogenerated electrons and effectively resist carrier recombination, which makes it an excellent electron transfer matrix. In addition, graphene oxide can act as a surfactant at the interface and reduce the energy between the interfaces due to the hydrophilic groups on the surface. In the photocatalytic process, the high‐energy electron–hole pairs generated by photoexcitation must resist recombination and also move from the bulk phase to the surface reactive sites to participate in the catalytic reaction. Nickel‐based compounds are considered promising nonprecious metal catalysts for photocatalytic water splitting for hydrogen production due to their low price and unique electronic structure.^[^ ^30^, ^31^ ^]^ The 3D structure helps to maximize the coverage of nickel on the surface of the photocatalyst, providing dense active sites and effectively increasing the surface reaction rate.

In this study, we focused on the design of a 3D hollow heterojunction in the presence of a strong built‐in electric field, as well as on the creation of additional electron transport channels and dense surface reaction sites to facilitate efficient charge separation, enabling the spatial separation of redox sites. Cu_2_O─S@GO@Zn_0.67_Cd_0.33_S 3D hollow heterojunctions were rationally constructed and photo deposited with a nonprecious metal for application in visible photocatalytic H_2_ production. The hydrogen production rate of the 7% Cu_2_O─S@GO@Zn_0.67_Cd_0.33_S–4.5 wt.% Ni(OH)_2_ nanohybrids reached 48.5 mmol g^−1^ h^−1^ (corresponding to an apparent quantum efficiency at 420 nm of 36.2%), which was 97 times higher than that of pure Zn_0.67_Cd_0.33_S. This significant improvement in performance was attributed to the formation of a p‐n heterojunction between Cu_2_O─S and Zn_0.67_Cd_0.33_S, which generated an electric field between the two components, thereby promoting the effective separation of photogenerated charges and increasing the electron transfer rate. The photothermal effect of Cu_2_O─S@GO and Ni(OH)_2_ increased the surface reaction rate, contributing to a significant improvement in photocatalytic performance. Finally, the mechanism of photocatalytic H_2_ production was elucidated in detail, providing a novel direction for the design of efficient visible light‐responsive artificial catalysts. In summary, this work investigates the design of 3D hollow nanostructures and the precise regulation of electric charges.

## 2 Results and Discussion

### 2.1 Material Characterizations

**Figure 1:**
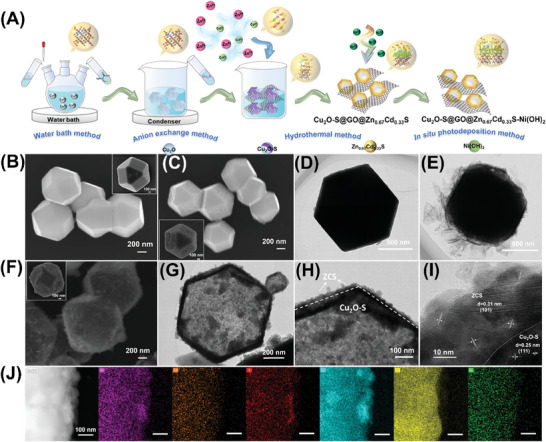
A) Synthesis and physicochemical properties of Cu_2_O─S@GO@Zn_0.67_Cd_0.33_S─Ni(OH)_2_ photocatalysts. SEM image of B) Cu_2_O and C) Cu_2_O─S. TEM of D) Cu_2_O─S and E) Cu_2_O─S@GO. F) SEM, G,H) TEM, and I) HRTEM images along with J) STEM and EDX elemental mappings of 7% CSGZCS‐4.5N.

As illustrated in **Figure** **1**A, Cu_2_O truncated rhombic dodecahedra was first obtained by thermostatic deposition. The oxygen ions on the Cu_2_O surface were then replaced by sulfur ions to obtain Cu_2_O─S via anion exchange. Subsequently, Cu_2_O─S was ultrasonically compounded with GO to form Cu_2_O─S@GO. The hollow Cu_2_O─S@GO@Zn_0.67_Cd_0.33_S (CSGZCS) 3D heterojunction was then hydrothermally synthesized using Cu_2_O─S@GO and Zn_0.67_Cd_0.33_S as precursors. L‐cysteine provided a source of sulfur that was sufficiently reductive to maintain the Cu_2_O state. Finally, Cu_2_O─S@GO@Zn_0.67_Cd_0.33_S─Ni(OH)_2_ (CSGZCS‐N) photocatalysts were obtained via in situ deposition of Ni ions under visible light irradiation. The crystalline structures of Cu_2_O and Cu_2_O─S were determined by X‐ray diffraction (XRD; Figure S1A, Supporting Information). The XRD patterns of Cu_2_O and Cu_2_O─S were identical, with a strong characteristic peak corresponding to the (111) plane (PDF No. 05–0667); no characteristic peaks of CuO were observed. The XRD peaks of Cu_2_O─S had a reduced intensity compared to those of Cu_2_O, indicating a decrease in crystallinity. As shown in Figure S1B (Supporting Information), the XRD pattern of Zn_0.67_Cd_0.33_S (ZCS) displayed six peaks at 2θ = 25.1°, 26.8°, 28.4°, 44.0°, 48.1°, and 52.1°, which could be assigned to the (100), (002), (101), (110), (103), and (112) planes of the hexagonal ferrite phase (JCPDS No. 40–0835), respectively.^[^ ^32^ ^]^ The XRD patterns of the CSGZCS heterojunction were largely in agreement with those of the ZCS crystal, with no detectable signals of Cu_2_O─S, indicating a low amount of this phase. To determine the ratio of Zn to Cd in Zn_0.6_7Cd_0.33_S, we carried out inductively coupled plasma–mass spectrometry (ICP–MS) tests. The Zn/Cd atomic ratio was calculated to be ≈1.97:1, which is generally consistent with the Zn_0.67_Cd_0.33_S composition. In addition, the specific content of Cu_2_O─S in the 7% Cu_2_O‐S@GO@Zn_0.67_Cd_0.33_S (7% CSGZCS) heterojunction was determined to be ≈6.79 wt.% by ICP‐MS. As shown in Figure S2A, Supporting Information, the characteristic Raman peaks of Cu_2_O and Cu_2_O─S were observed at 149, 200, 218, 405, and 630 cm^−1^.^[^ ^33^ ^]^ The Raman spectrum of 7% CSGZCS showed characteristic peaks at 212 and 406 cm^−1^ (Figure S2A, Supporting Information), which were not observed in the spectrum of pure ZCS, indicating the presence of Cu_2_O─S in the heterojunction.

The morphologies of the synthesized samples were characterized by scanning electron microscopy (SEM) and transmission electron microscopy (TEM). As shown in Figure 1B and S3a, pristine Cu_2_O showed a homogeneous rhombic dodecahedron cross‐sectional morphology with a smooth surface, a diameter of ≈800 nm, and mainly exposed (111) and (100) faces. The corresponding elemental mapping revealed a relatively uniform distribution of Cu and O elements (Figure S3B, Supporting Information). As shown in Figure 1C and Figure S3C (Supporting Information), the Cu_2_O─S surface became rough after vulcanization, and the oxygen ions on the (100) face showed a higher tendency to be substituted by sulfur ions.^[^ ^34^, ^35^ ^]^ The (111) crystal faces with suspended Cu atoms were protected by the negative charge, while the neutral (100) crystal faces were less protected. As a result, the rate of the Kirkendall process differed between the S and O atoms on the different crystalline planes. However, due to the limited substitution of O by S atoms, the elemental mapping was dominated by Cu and O, without any significant contribution of S (Figure S3D, Supporting Information). Upon the addition of more sulfur ions, the surface of Cu_2_O─S/2 adopted an irregular sawtooth shape (Figure S3E, Supporting Information), and the elemental mapping confirmed the presence of S (Figure S3F, Supporting Information). The corresponding energy‐dispersive X‐ray spectroscopy (EDX) profiles in Figure S4 (Supporting Information) show an increased S content in Cu_2_O─S/2 compared to Cu_2_O─S. Thus, as the amount of added sulfur ions increased, more oxygen ions could be substituted. Based on the TEM images in Figure 1D, the planes of Cu_2_O─S had a hexagonal shape; moreover, the high‐resolution TEM (HRTEM) image in Figure S5 Supporting Information shows a lattice fringe pattern corresponding to Cu_2_O─S. The interplanar distance was 0.25 nm, which could be attributed to the (111) plane of Cu_2_O─S.^[^ ^36^ ^]^ The TEM image of Cu_2_O─S@GO in Figure 1E and Figure S6 (Supporting Information) shows that an ultrathin sheet of GO was successfully covered around the surface of Cu_2_O─S, which facilitates the interpolation of GO nanosheets between Cu_2_O─S and ZCS.

As shown in Figure 1F, ZCS was uniformly coated on the surface of the Cu_2_O─S dodecahedron and maintained its original phase after loading Ni. The inset shows that Cu_2_O─S@GO@Zn_0.67_Cd_0.33_S─Ni(OH)_2_ (CSGZCS‐N) had a 3D structure. Pure ZCS grew in situ on the Cu_2_O─S dodecahedral surface in the form of inhomogeneous microspheres (Figure S7, Supporting Information). Hollow Cu_2_O─S@GO@Zn_0.67_Cd_0.33_S 3D structures were obtained based on the Kirkendall effect (Figure S8, Supporting Information).^[^ ^37^ ^]^ It can be assumed that the reducing environment provided by L‐cysteine is more favorable for the Cu_2_O nanocrystals to maintain their steric structure. As indicated by the TEM images in Figure 1G,H, the CSGZCS‐N nanohybrids essentially maintained their hollow 3D structures after the photo deposition of Ni. The rough surface showed a large number of atomic steps, which provided a higher number of exposed active sites in the CSGZCS‐N nanohybrids. Above the white dashed line, ZCS grew close to the surface of Cu_2_O, as shown in Figure 1H. The lattice spacing of 0.31 nm was assigned to the (101) plane of ZCS,^[^ ^37^ ^]^ while the 0.27 nm spacing corresponded to the (100) face of Ni(OH)_2_ (Figure 1H).^[^ ^22^ ^]^ In addition, Figure 1I clearly shows a fringe spacing of 0.25 nm, which could be attributed to the (111) face of Cu_2_O─S. This also provides evidence for the successful encapsulation of ZCS on the Cu_2_O─S surface. The scanning TEM (STEM) mapping images in Figure 1J show that Zn, Cd, S, Cu, O, and Ni were uniformly dispersed on the polyhedra. The above results confirm the successful preparation of the CSGZCS 3D structure and the homogeneous loading of Ni as a co‐catalyst.

### 2.2 Origin of Enhanced Photocatalytic Activity

**Figure 2:**
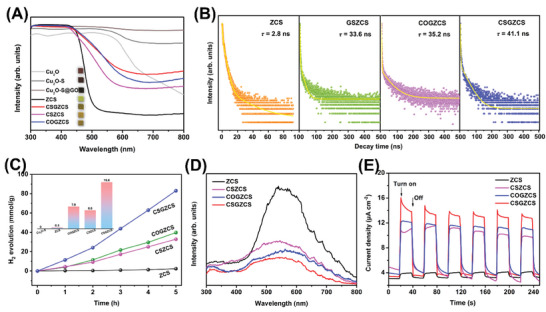
A) UV–vis DRS spectra of the different photocatalysts. B) TRPL decay spectra, C) Comparison of photocatalytic H_2_ evolution activities, D) PL spectra, and E) transient photocurrent response spectra of the ZCS, 7% CSZCS, 7% COGZCS, and 7% CSGZCS samples.

The optical properties of the catalysts were investigated by UV–vis diffuse reflectance spectroscopy (UV–vis DRS), as shown in **Figures** **2**A and S9 (Supporting Information). ZCS responded to visible light and exhibited a visible light absorption edge at ≈540 nm, corresponding to an intrinsic bandgap (E_g_) of 2.67 eV (Figure S10A, Supporting Information). In comparison, the visible light absorption of sulfated Cu_2_O─S was significantly enhanced, and the absorption band edge was red‐shifted. The 7% Cu_2_O‐S@GO@Zn_0.67_Cd_0.33_S (7% CSGZCS) composite synthesized from the Cu_2_O─S@GO precursor showed stronger light absorption than the 7% Cu_2_O@GO@Zn_0.67_Cd_0.33_S (7% COGZCS) composite. The calculated bandgap of Cu_2_O─S (1.95 eV) was narrower than that of Cu_2_O (2.04 eV; Figure S10B,C, Supporting Information). Figure S8 (Supporting Information) shows that the light absorption capacity of the catalysts increased with increasing Cu_2_O─S content. Interestingly, the type of precursor significantly affected the light absorption capacity of the composite. The 7% Cu_2_O‐S@Zn_0.67_Cd_0.33_S (7% CSZCS) composite exhibited weaker light absorption than the 7% CSGZCS counterpart, indicating that the addition of GO improved the light absorption (Figure 2A). Therefore, the addition of Cu_2_O─S and GO enhanced the light absorption as well as expanded the visible light absorption range. The average carrier lifetimes of the catalysts were examined by time‐resolved photoluminescence (TRPL) decay spectroscopy, as shown in Figure 2B (see Table S2, Supporting Information for the kinetic calculations based on TRPL decay spectra). The average charge lifetimes of ZCS, CSZCS, COGZCS, and CSGZCS were 2.76, 33.6, 35.2, and 41.1 ns, respectively. A longer lifetime reflects more opportunities to participate in surface reactions, which correspond to a higher photocatalytic efficiency.^[^ ^31^ ^]^ Compared with pure ZCS, the CSGZCS heterojunction had a longer lifetime of photogenerated carriers, and could thus provide more photoelectrons for photocatalytic reduction.

The visible light‐catalyzed H_2_ evolution efficiencies of the catalysts are shown in Figure 2C. Pure ZCS had a low H_2_ evolution rate (0.5 mmol g^−1^ h^−1^), which could be attributed to the high complexation rate of photogenerated carrier. The absence of visible‐light‐driven H_2_ production in Cu_2_O─S may be due to the severe photocorrosion of Cu_2_O. As shown in Figure S11 (Supporting Information) we optimized the Cu_2_O─S content in the heterojunction. The highest photocatalytic H_2_ production activity of 16.6 mmol g^−1^ h^−1^ was achieved by the 7% CSGZCS heterojunction. As shown in Figure 2C, the 7% COGZCS and 7% CSZCS catalysts exhibited lower H_2_ evolution efficiencies than 7% CSGZCS. Thus, the addition of Cu_2_O─S and GO effectively improved the photocatalytic H_2_ production. As shown in Figure 2D, bare ZCS showed the strongest PL peak at ≈540 nm; this can be attributed to charge recombination and bandgap jumping.^[^ ^38^ ^]^ The fluorescence intensity of the CSZCS heterojunction was significantly reduced compared with that of pure ZCS, indicating that Cu_2_O─S effectively improved the charge separation. Importantly, among the tested catalysts, CSGZCS had the lowest PL peak intensity, corresponding to the lowest recombination rate of photogenerated carriers. The experimental results indicate that the synergistic effect of Cu_2_O─S and GO effectively improved the separation efficiency of photogenerated charges and prevented their recombination. The enhanced charge transfer of the CSGZCS catalyst was further examined through photocurrent response (*I–t*) curves and electrochemical impedance spectroscopy (EIS) spectra (Figure 2E; Figure S12, Supporting Information). The higher photocurrent and smaller impedance arc radius of CSGZCS compared to the other catalysts indicate a higher charge transfer rate, longer charge lifetime, and significantly enhanced photocatalytic hydrogen evolution performance.

### 2.3 Precise Regulation of Surface Charge Separation

**Figure 3:**
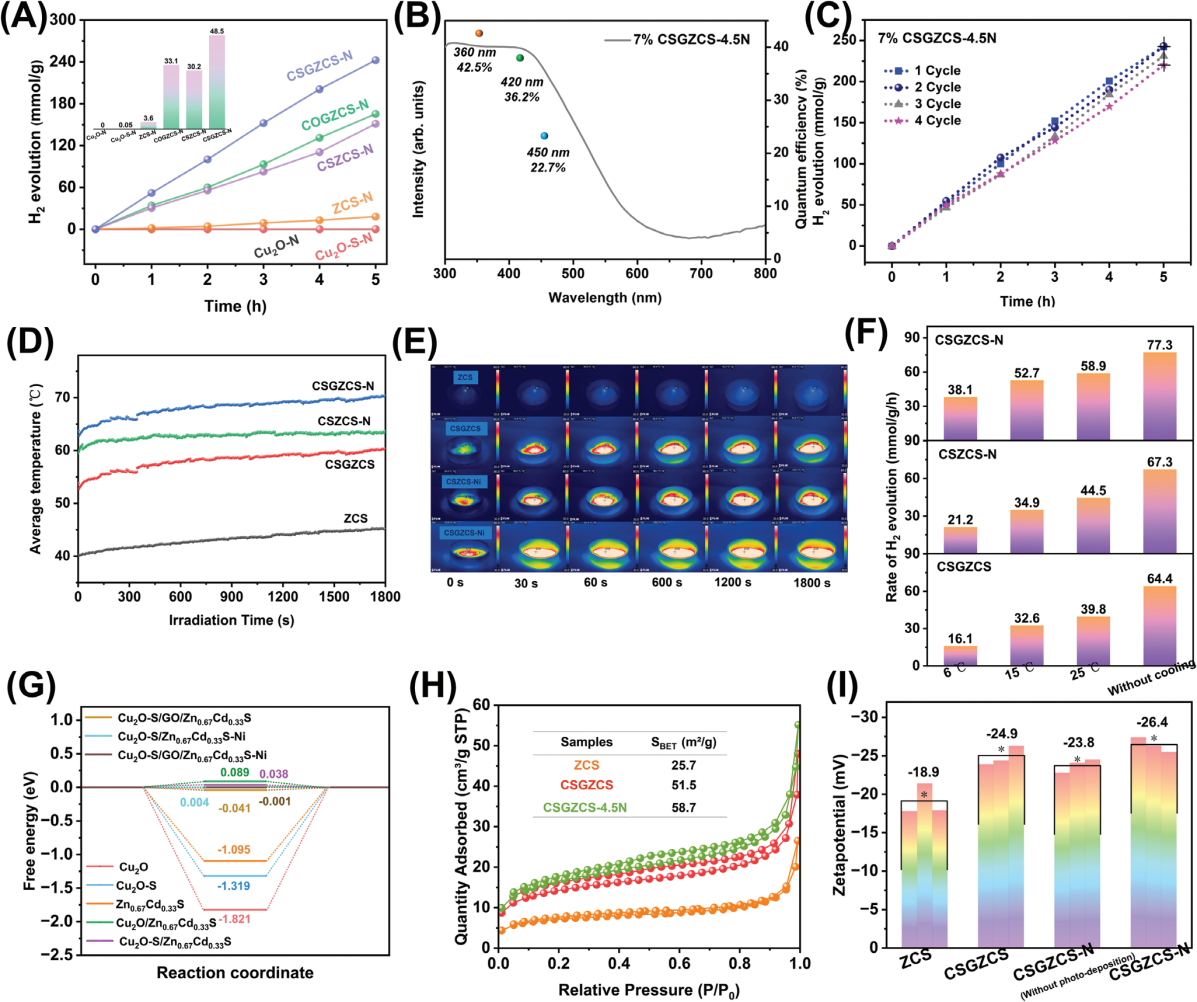
A) Comparison of photocatalytic H_2_ evolution activities of different composites. B) Apparent quantum efficiencies of 7% CSGZCS‐4.5N under monochromatic light irradiation at various wavelengths (360, 420, and 450 nm). C) Cycling performance of 7% CSGZCS‐4.5N. D) Average temperatures of different photocatalysts as a function of time during light irradiation. E) Infrared thermographic images of different photocatalysts. F) H_2_ evolution rates of 7% CSGZCS, 7% CSZCS‐4.5N, and 7% CSGZCS‐4.5N at different external condensation temperatures and without cooling. G) Free energy diagrams for HER on the surfaces, H) Nitrogen physisorption isotherms, and I) Zeta‐potential of different photocatalysts.

In order to further improve the surface reaction rate and precisely regulate the charge flow, Ni^2+^ was loaded on the photocatalyst surface as a co‐catalyst to investigate its performance in photocatalytic hydrogen evolution. As shown in **Figure** **3**a, the photocatalytic hydrogen evolution rate of Cu_2_O─N still displayed no activity. Compared with Cu_2_O─S (rate = 0), the photocatalytic hydrogen evolution of Cu_2_O─S─N reached 0.05 mmol g^−1^ h^−1^, indicating that Ni^2+^, serving as the active site, suppresses the complexation of photogenerated carriers. The hydrogen production rate of Zn_0.67_Cd_0.33_S‐Ni(OH)_2_ (ZCS‐N) reached 3.6 mmol g^−1^ h^−1^, which was 7.2 times that of ZCS (0.5 mmol g^−1^ h^−1^). Similarly, the hydrogen evolution rates of the COGZCS and CSZCS heterojunctions showed substantial increases after the photodeposition of Ni^2+^. Surprisingly, upon loading with 4.5 wt.% Ni^2+^ as cocatalyst, the 7% Cu_2_O‐S@GO@Zn_0.67_Cd_0.33_S‐4.5wt.%Ni(OH)2 (7% CSGZCS‐4.5N) exhibited a high H_2_ yield of 48.5 mmol g^−1^ h^−1^, ≈97 times that of pure ZCS. This indicates that the Ni^2+^ cocatalyst was uniformly deposited on the catalyst surface during the photocatalytic process, which reduced the surface reaction activation energy and increased the charge separation rate. As shown in Figure S13 (Supporting Information), the photocatalytic hydrogen evolution performance tended to increase and then decrease with increasing Ni^2+^ loading; in particular, the gradual increase led to a significant decrease in activity. This is due to the fact that an excessive Ni^2+^ loading leads to a blocked light absorption on the catalyst surface, which reduces the amount of photoelectrons in the heterojunction and contributes to a significant decrease in photocatalytic activity. Compared to the catalysts loaded with 4.5 wt.% Ni^2+^, those loaded with 4.5 wt.% Pt exhibited a worse H_2_ production performance, suggesting that Ni is more effective in promoting the directional transfer of photogenerated electrons to enhance H_2_ production. As shown in Figure S14 (Supporting Information), the hydrogen evolution performance of Cu_2_O─S@GO@ZCS‐Ni(OH)_2_ is slightly affected by different theoretical contents of GO. The performance of the composite catalysts was slightly decreased when the GO addition reached a certain level, which was attributed to the fact that too much GO would affect the contact between the interface of ZCS and Cu_2_O─S heterojunction. The apparent quantum efficiency of 7% CSGZCS‐4.5N was measured under monochromatic light irradiation at different wavelengths (Figure 3B). The AQE under irradiation at 420 nm was 36.2%. The performances of the heterojunctions with 3D morphology are compared in Figure S15, Table S3 (Supporting Information). The results confirmed that the rational construction of p‐n junctions and the modulation of the 3D hollow structure successfully achieved the spatial separation of the photogenerated charges. The stability of the photocatalyst was evaluated by cycling tests, and the results are shown in Figure 3C. Compared with the first‐cycle value, the H_2_ evolution rate after the fourth cycle (15 h) remained at ≈92.1%, which highlighted the relative stability of 7% CSGZCS‐4.5N. In addition, the XRD patterns of 7% CSGZCS were recorded before and after the cycling tests, and the results are shown in Figure S16A (Supporting Information). The comparison shows that the crystal structure of 7% CSGZCS remained basically unchanged, but its crystallinity decreased significantly and a new peak corresponding to Ni(OH)_2_ appeared at ≈20.5°; this confirmed the in situ photodeposition of the Ni cocatalyst in the form of Ni(OH)_2_.^[^ ^22^, ^39^ ^]^ In addition, the morphological analysis of the reacted 7% CSGZCS found no changes, indicating the strong stability of the 3D structure of the catalyst, as shown in Figure S16B (Supporting Information).

To confirm the photothermal coupling effect, the temperatures of the ZCS, CSGZCS, CSZCS‐N, and CSGZCS‐N photocatalysts were recorded during light irradiation. Figure 3D shows the average temperature variation of the catalysts during light irradiation. The average temperature increased rapidly within the first 5 min of irradiation, then increased slowly and reached equilibrium within 30 min. Detailed images of the temperature change with light exposure time are shown in Figure 3E (see the IR thermography video in Figure S17, Supporting Information for details). The temperature of ZCS reached 42.5 °C after 30 min of irradiation, whereas the temperatures of CSGZCS (58.2 °C), CSZCS‐N (61.5 °C), and CSGZCS‐N heterojunction (70.6 °C) were significantly higher. These results indicate that the addition of Cu_2_O─S and GO synergistically enhanced the photothermal effect of ZCS, and the photodeposited Ni also resulted in increased light absorption, which further improved the photothermal effect. Figure 3F shows the H_2_ evolution rates of CSGZCS, CSZCS‐N, and CSGZCS‐N photocatalysts at different external condensation temperatures and without cooling. The hydrogen evolution rates of the different heterojunctions increased with increasing cooling temperature, consistent with the trend of the photothermal effect.^[^ ^11^, ^40^ ^]^ It is worth mentioning that the activity of the CSGZCS‐N heterojunction reached 77.3 mmol g^−1^ h^−1^ without cooling.

Because the hydrogen evolution reaction (HER) involves the adsorption of H atoms on the catalyst surface and the recombinative desorption of molecular hydrogen from the surface, the HER activity is closely correlated to the adsorption strength of H atom. A hydrogen adsorption‐free energy (|Δ*G*_H*_|) close to zero implies a high catalytic activity for the HER.^[^ ^41^, ^42^ ^]^ Figure 3G and Figures S18 and S19 (Supporting Information) show the H adsorption Gibbs free energies of various heterojunctions and the corresponding construction models. Although the Cu_2_O/Cu_2_O─S and Zn_0.67_Cd_0.33_S catalysts had highly negative Δ*G*_H*_ values of −1.821/−1.319 and −1.095 eV, respectively, the excessively strong H adsorption would hinder the desorption of H_2_ from the catalyst surface, leading to poor catalytic activity. In contrast, the Δ*G*_H*_ value of the Cu_2_O/Zn_0.67_Cd_0.33_S heterojunction (0.089 eV) was much higher than those of its components, indicating a significantly enhanced catalytic activity, which might be due to the synergistic effect and charge redistribution between Cu_2_O and Zn_0.67_Cd_0.33_S. Upon substituting an interfacial O atom of Cu_2_O in Cu_2_O/Zn_0.67_Cd_0.33_S by an S atom, the Δ*G*_H*_ of the formed Cu_2_O─S/Zn_0.67_Cd_0.33_S heterojunction was slightly reduced to 0.038 eV, indicating an improved HER activity. Interestingly, the Δ*G*_H*_ value (0.004 eV) of the Cu_2_O‐S/Zn_0.67_Cd_0.33_S─Ni heterojunction is close to zero, and the corresponding Δ*G*_H*_ value (−0.001) of the Cu_2_O─S/GO/Zn_0.67_Cd_0.33_S─Ni is almost zero, which further illustrates that the heterojunctions theoretically exhibit much higher catalytic performance than the components. Although the overpotential did not change after the addition of GO, the Δ*G*_H*_ for H adsorption all became negative, indicating that the first H adsorption before the decisive velocity step becomes a desorption process with the formation of H_2_ after the second H adsorption. Note that the HER catalytic activity of the as‐designed Cu_2_O─S/GO/Zn_0.67_Cd_0.33_S─Ni heterojunction was superior to the activities of previously reported catalysts.^[^ ^43^ ^]^ In addition, as shown in Figures S20 and S21 (Supporting Information), the H atom adsorption at the bridge site between Zn and Cd/Ni atoms was stronger than that at other sites, indicating that the Zn─Cd/Ni bridge site was the most favorable active site for the HER. Bader charge analysis shows that the adsorbed H atoms gain 0.708/0.536 e from the connected Zn and Cd/Ni atoms (compared to 0.318/0.284 e obtained without GO), which enables the H atoms to be stably adsorbed on the Zn─Cd/Ni bridge sites and favors a larger number of electrons for the H atoms. This suggests that the photogenerated electrons are first captured by the surface Zn and Cd/Ni atoms and then react with H. The exact Bader charge also proves that the H adsorption is more stable after the addition of GO.

It is well known that the larger specific surface area of photocatalysts can provide abundant active sites that facilitate photocatalytic reactions.^[^ ^44^, ^45^ ^]^ Therefore, surface areas were evaluated from nitrogen adsorption–desorption isotherms. As shown in Figure 3H, all samples showed typical type IV isotherms indicating their mesoporous structure, and the inset displays the specific Brunauer–Emmett–Teller surface area (SBET) values. Furthermore, the SBET of CSGZCS (51.5 m^2^ g^−1^) was larger than that of pure ZCS (25.7 m^2^ g^−1^), indicating that the construction of the 3D hollow heterojunction was beneficial to increase the surface area. In addition, after the photodeposition of Ni on the catalyst surface, the specific surface area of Cu_2_O─S@GO@Zn_0.67_Cd_0.33_S─Ni(OH)_2_ (CSGZCS‐N) showed a further increase, providing abundant surface active sites for the reduction reaction. Moreover, the pore size distribution confirmed the mesoporous structure of the catalyst (Figure S22, Supporting Information). Compared with ZCS, both the construction of the 3D hollow heterojunction and the surface photodeposition of Ni^2+^ effectively increased the pore size, indicating the creation of more electron migration channels that facilitated the spatial separation of charges.

To investigate the physical location of the surface‐photodeposited Ni^2+^ species, Zeta potential analysis was performed for ZCS, CSGZCS, and CSGZCS‐N catalysts, and the results are shown in Figure 3I. Compared with ZCS (−18.9 mV), the surface zeta potential of the CSGZCS heterojunction was more negative (−24.9 mV), indicating that the surface of the heterojunction had a more negative charge. Ni^2+^ ions were uniformly adsorbed on the surface of the heterojunction by ultrasonication, and the surface potential of the obtained CSGZCS‐N without photodeposition was slightly reduced, indicating that Ni^2+^ was adsorbed on the heterojunction surface by electrostatic interactions. In addition, after photodeposition the surface potential of the CSGZCS‐N heterojunction became more negative, indicating that Ni gained access to electrons as active sites.

**Figure 4:**
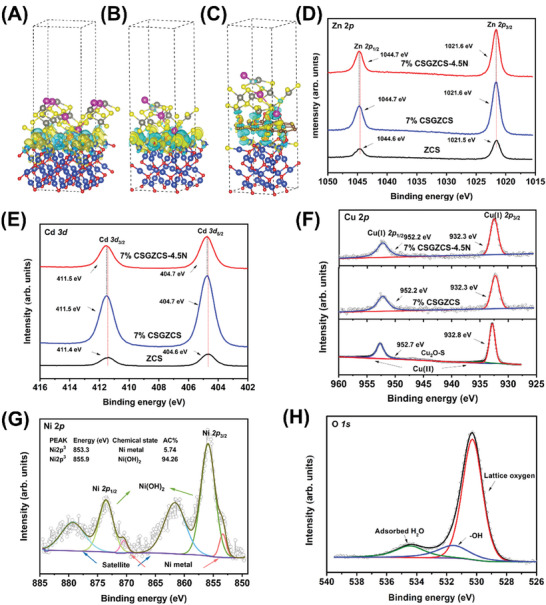
Charge density difference maps of A) Cu_2_O─S/Zn_0.67_Cd_0.33_S, B) Cu_2_O─S/Zn_0.67_Cd_0.33_S─Ni, and C) Cu_2_O─S/GO/Zn_0.67_Cd_0.33_S─Ni heterojunctions. The yellow and green regions denote electron accumulation and depletion, respectively, and the isosurface value is set to 0.003 *e*/bohr.^3^ D) Cu 2p spectra of Cu_2_O─S, 7% CSGZCS, and 7% CSGZCS‐4.5N. E) Zn 2p and F) Cd 3d XPS spectra of ZCS, 7% CSGZCS, and 7% CSGZCS‐4.5N. G) Ni 2p spectra (The inset is a semi‐quantitative XPS analysis), and H) O 1s spectra of 7% CSGZCS‐4.5N.

To determine the potential reaction mechanism of the Cu_2_O─S/Zn_0.67_Cd_0.33_S heterojunction, charge density differences were evaluated by density functional theory calculations. The good saturation of dangling bonds enabled the formation of the desired interface between Cu_2_O/Cu_2_O─S and Zn_0.67_Cd_0.33_S (Figures S23 and S24, Supporting Information), which is a prerequisite for a high interfacial charge transfer efficiency.^[^ ^46^ ^]^ In **Figure** **4**A,B, the yellow regions around the doped S atom and the O atoms on the Cu_2_O─S surface represent charge accumulation, while the green regions around the metal atoms (Cu/Zn/Cd) denote charge depletion at the Cu_2_O─S/Zn_0.67_Cd_0.33_S heterojunction interface. This charge transfer provides insights into how the interaction between the two semiconductors can achieve bonding. The strong electron coupling between Cu_2_O─S and Zn_0.67_Cd_0.33_S implies the presence of a tight electron transport interface in the heterojunction, which is beneficial for charge transfer and essential for obtaining excellent photocatalytic activity.^[^ ^46^, ^47^ ^]^ In addition, we also explored the Ni doping on the surface of the Cu_2_O─S/Zn_0.67_Cd_0.33_S heterojunction (Figure S18, Supporting Information). The results show that Cu_2_O─S/Zn_0.67_Cd_0.33_S─Ni exhibits a similar geometrical and electronic structure to that of the heterojunction without photo‐burdened Ni. The charges of the doped S atoms of Cu_2_O─S/Zn_0.67_Cd_0.33_S and Cu_2_O─S/Zn_0.67_Cd_0.33_S─Ni are 0.663 and 0.653 e, which are much lower than that of the substituted O atoms of Cu_2_O/Zn_0.67_Cd_0.33_S (1.039 e). Therefore, more electrons are involved in the subsequent H adsorption process on the Zn_0.67_Cd_0.33_S side of the heterojunction. Figure 4C shows the charge density distribution of Cu_2_O─S/GO/Zn_0.67_Cd_0.33_S─Ni with the addition of GO in the heterojunction. Interestingly, the Bader charge analysis reveals that the electrons lost by Zn_0.67_Cd_0.33_S with the addition of GO are 1.56 e compared to 2.23 e without GO, which inversely proves that more electrons are retained by the HER on the Zn_0.67_Cd_0.33_S side with the addition of GO.

To verify the above theoretical calculations, X‐ray photoelectron spectroscopy (XPS) was used to evaluate the chemical valence states and elemental composition of the catalysts. As shown in Figure S25A (Supporting Information), the Cu 2p spectrum of Cu_2_O─S showed high binding energy peaks corresponding to the Cu(I) 2p_3/2_ and Cu(I) 2p_1/2_ spin‐orbit components at 932.4 and 952.3 eV, respectively (932.2 and 952.4 eV for Cu_2_O).^[^ ^48^, ^49^ ^]^ A slight shift of the Cu(I) 2p peak toward lower binding energies was observed during the vulcanization process, indicating that S─Cu(I)─O bonds replaced the Cu(I)─O bonds.^[^ ^37^ ^]^ A pair of recombination satellite peaks with high binding energies were also detected in the spectra of Cu_2_O─S and Cu_2_O, indicating the presence of Cu(II) oxides. As shown in Figure S25B (Supporting Information), the O 1s peak of Cu_2_O─S was found at 531.9 eV. The slightly lower energy of this peak compared to the corresponding peak in Cu_2_O (532.0 eV) demonstrated the presence of S in the Cu_2_O lattice.^[^ ^49^ ^]^ The binding energy of the O 1s peak of lattice oxygens in Cu_2_O─S was found at 530.3 eV, a significantly lower energy than that of the corresponding Cu_2_O peak; this indicates the formation of the S─Cu(I)─O bonds. The chemical state of the S atoms in Cu_2_O─S was elucidated via the S 2p XPS spectrum (Figure S25C, Supporting Information), which showed binding energies of 161.8 and 162.9 eV for S 2p_1/2_ and 2p_3/2_, respectively. The observed S 2p binding energy of 168.7 eV was attributed to the presence of sulfate functional groups on the surface of Cu_2_O─S.^[^ ^37^ ^]^ The above XPS results show that the O atoms on the surface of Cu_2_O were partially replaced by S atoms, resulting in the formation of Cu_2_O─S while maintaining the original morphology of Cu_2_O.

Zn, Cd, S, Cu, O, and Ni elements were detected in 7% CSGZCS‐4.5N (Figure S26A, Supporting Information). The peaks at 1021.6 and 1044.7 eV in Figure 4D were assigned to Zn 2p_3/2_ and Zn 2p_1/2_, respectively, indicating the presence of the Zn(II) state in 7% CSGZCS‐4.5N. As shown in Figure 4E, the binding energies of Cd 3d in 7% CSGZCS‐4.5N were 404.7 and 411.5 eV, respectively.^[^ ^50^ ^]^ The S 2p XPS spectrum showed two peaks corresponding to S^2−^ at binding energies of 162.2 and 163.5 eV (Figure S26B, Supporting Information). The peaks at binding energies of 167.2 and 168.7 eV were attributed to sulfate functional groups on the surface. The Cu 2p_3/2_ and Cu 2p_1/2_ binding energies of 7% CSGZCS‐4.5N were 952.2 and 932.3 eV, respectively, which were lower than the binding energy of Cu(I) in Cu_2_O─S (Figure 4F).^[^ ^37^ ^]^ As shown in Figure 4A,B, compared with ZCS, the Zn 2p and Cd 3d binding energies in 7% CSGZCS showed a significant shift toward higher energy levels. In addition, the Cu 2p signal of 7% CSGZCS was clearly observed to shift toward lower binding energies in Figure 4F, and the characteristic peak of Cu(II) disappeared. This indicates the occurrence of a significant electron transfer between Cu_2_O─S and ZCS upon contact. It should be noted that changes in elemental binding energy can directly reflect variations in electron density. Thus, the changes in binding energy determine the direction of interfacial charge transfer in the heterojunction. This suggests that ZCS tends to transfer charge to Cu_2_O─S through the interface when the photocatalyst is not irradiated,^[^ ^23^ ^]^ which is consistent with the above theoretical results. As shown in Figure 4G, the Ni 2p spectrum included a pair of peaks with binding energies of 855.9 and 873.5 eV, corresponding to Ni 2p_3/2_ and Ni 2p_1/2_, respectively. A second pair of satellite peaks of Ni 2p appeared at 861.6 and 879.9 eV.^[^ ^51^ ^]^ In addition, two prominent satellite peaks were observed at 861.6 and 879.9 eV, probably due to the excitation of unpaired 3D electrons to higher binding energy levels.^[^ ^52^ ^]^ In addition, a pair of peaks at 853.3 and 870.4 eV corresponded to Ni 2p_3/2_ and Ni 2p_1/2_ of Ni metal, respectively. Semi‐quantitative XPS analysis of Ni 2p^3^ showed that Ni(OH)_2_ accounted for 94.3% and Ni metal for ≈5.7%. Figure 4H showed the O 1s peak of 7% CSGZCS‐4.5N was located at 530.3, 531.5, and 534.2 eV, corresponding to the lattice oxygens of Cu(I)─O, hydroxyl oxygen (Ni─OH) and adsorbed H_2_O, respectively. The content of Ni in 7% CSGZCS‐4.5N was determined by ICP–MS to be 0.49 wt.%. In the Cu *LMM* spectrum (Figure S26, Supporting Information), the peak at 571.8 eV was identified as the Cu^+^ state in Cu_2_O─S.^[^ ^53^ ^]^ The above results basically indicate that the construction of the heterojunction effectively suppressed the photocorrosion of Cu_2_O─S.

### 2.4 Photocatalytic Mechanism

**Figure 5:**
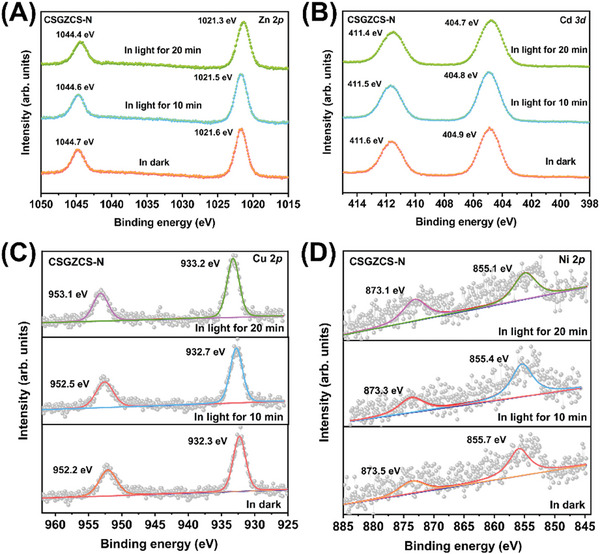
In situ A) Zn 2p, B) Cd 3d, C) Cu 2p, and D) Ni 2p XPS spectra of CSGZCS‐N.

To further investigate the transfer of interfacial charges in the heterojunctions during photocatalysis, we performed in situ XPS measurements. Notably, the changes in binding energy can be used to detect the direction of carrier transfer in a heterojunction.^[^ ^44^, ^54^, ^55^ ^]^ As shown in **Figure** **5**A, the binding energy of Zn 2p electrons in CSGZCS‐N shifted slightly (0.1 eV) toward lower values at 10 min of light irradiation, indicating an increase in electron density. After continued light irradiation for 10 min, the Zn 2p binding energy continued to shift to lower values and the electron density further increased. At the same time, the Cd 3d binding energy also gradually shifted to lower values with increasing irradiation time, as shown in Figure 5B.

This implies that the increase in electron density at the interface due to the electrons gained by ZCS under illumination indicated a decrease in binding energy. In contrast, compared to CSGZCS‐N in dark conditions, the binding energy of Cu 2p electrons in CSGZCS‐N markedly shifted to higher values under light irradiation (Figure 5C). More importantly, as shown in Figure 5D, the Ni 2p binding energy significantly shifted to lower values, suggesting that the Ni atoms on the catalyst surface gained electrons and the electron density increased. Therefore, these XPS results provide important insight into the carrier transfer pathway at the CSGZCS‐N heterojunction interface under light irradiation. In particular, the migration of photogenerated electrons from Cu_2_O─S to ZCS and then further to Ni on the photocatalyst surface is a sufficient condition to achieve the spatial separation of photogenerated carriers in the heterojunction. Most interestingly, the in situ XPS results matched perfectly with the above zeta potential test results.

**Figure 6:**
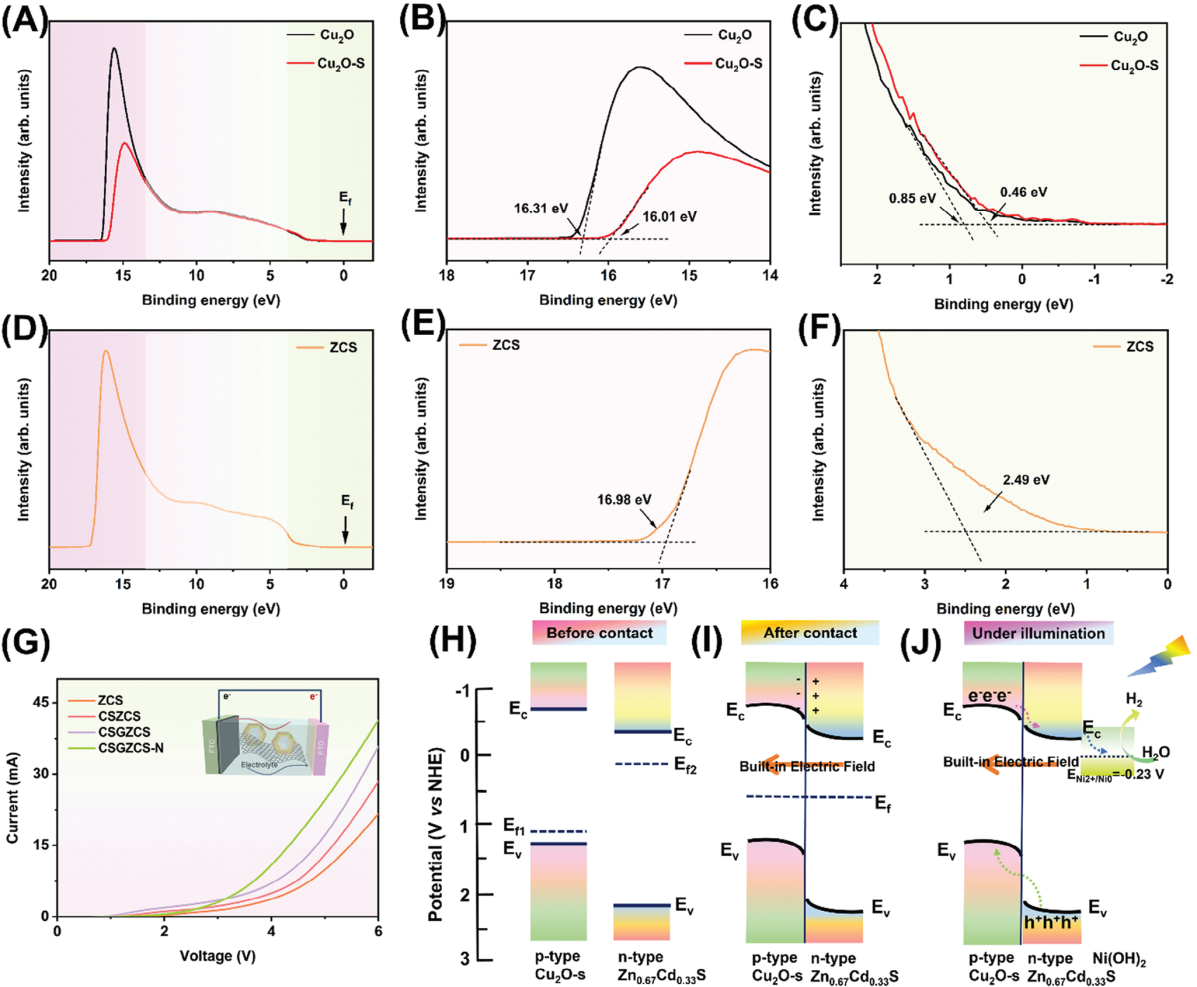
UPS spectra of A–C) Cu_2_O and Cu_2_O─S, and D–F) ZCS. G) I‐V curves of ZCS, CSZCS, CSGZCS and CSGZCS‐N. H–J) Schematic energy level diagrams of Cu_2_O─S@GO@Zn_0.67_Cd_0.33_S─Ni(OH)_2_ p‐n junction formation.

The energy bands of the samples were analyzed by ultraviolet photoelectron spectroscopy (UPS).^[^ ^56^, ^57^ ^]^ As shown in **Figure** **6**A−F, upon referencing the optical emission spectrum to the Fermi level, the typical secondary electron cutoff energies of Cu_2_O, Cu_2_O─S, and ZCS were 16.31, 16.01, and 16.98 eV, respectively. The work functions (*Φ*) of Cu_2_O, Cu_2_O─S, and ZCS were determined to be −4.89, −5.19, and −4.22 eV, respectively, based on the difference between the photon energy (21.2 eV) and the cutoff energy. In addition, further linear extrapolation along the tangent line to the initial part of the spectrum shows that the Fermi energy level maxima (E_VB_ − E*_f_*) of Cu_2_O, Cu_2_O─S, and ZCS were −0.85, −0.46, and −2.49 eV, respectively. Thus, the valence band (VB) positions of Cu_2_O, Cu_2_O─S, and ZCS were 1.24, 1.15, and 2.21 eV versus normal hydrogen electrode (NHE), respectively. As shown in Figure S10 (Supporting Information), the bandgap values of Cu_2_O, Cu_2_O─S, and ZCS were 2.04, 1.95, and 2.67 eV, respectively. Combining the above results, the conduction band (CB) positions of Cu_2_O, Cu_2_O─S, and ZCS were estimated to be −0.8, −0.8, and −0.46 eV versus NHE, respectively.

In order to determine the type and electronic structure properties of the semiconductor, we obtained Mott–Schottky (M–S) curves.^[^ ^58^ ^]^ As shown in Figure S28 (Supporting Information), the M–S curve of ZCS had a positive slope, indicating that the catalyst exhibited the characteristics of an n‐type semiconductor. On the other hand, the M–S curve of Cu_2_O─S had a negative slope, indicating a p‐type semiconductor. When p‐type ZCS formed a heterojunction with n‐type Cu_2_O─S, a strong internal electric field could be formed at the interface, due to the positive charge of the main carriers of the p‐type semiconductor and the negative charge of the n‐type heterojunction, which further enhanced the separation and transfer of photoinduced carriers at the interface. In addition, the flat‐band potentials (E*_f_*) of ZCS and Cu_2_O─S were −0.28 and 0.69 eV (relative to the NHE) at pH 0, which are consistent with the Fermi energy level estimated from the UPS spectrum. As shown in Figure 6G, the rectification characteristics of the device were tested by testing the carrier transport characteristics (*I*–*V*) in the presence of a tighter and larger interface, which also indicates a gradual enhancement of the built‐in electric field.^[^ ^59^ ^]^

Figure 6H shows the energy level diagrams of Cu_2_O─S and Zn_0.67_Cd_0.33_S. The electron transfer mechanism between Cu_2_O─S and ZCS p‐n heterojunctions in the absence of light is shown in Figure 6I. When ZCS and Cu_2_O─S are in close contact, the electrons of ZCS are transferred to Cu_2_O─S through the interface until their Fermi energy levels are aligned. As a result, ZCS loses electrons and becomes positively charged. At the same time, a space charge region is formed on one side of ZCS, leading to an upward bending of the energy band.^[^ ^25^ ^]^ At the same time, a downward bending of the energy band occurs on the Cu_2_O─S side, due to the electron transfer to it. Figure 6J shows the electron and hole transfer mechanism when Cu_2_O─S and ZCS were in contact to form a heterojunction. Under light irradiation, electrons in the conduction band of Cu_2_O─S are transferred to the conduction band of ZCS. As the Ni^2+^/Ni^0^ potential value is −0.23 eV, the photoelectrons in the conduction band of ZCS tend to migrate toward Ni(OH)_2_,^[^ ^30^, ^51^ ^]^ and the photogenerated holes of ZCS migrate toward the VB of Cu_2_O─S. Thus, the Cu_2_O─S@GO@Zn_0.67_Cd_0.33_S─Ni(OH)_2_ nanohybrids provide a mechanism for the spatial separation of photogenerated carriers.

In summary, the 3D hollow structure facilitates the generation of an internal electric field at the interface of the two components, creating more migration channels through the tighter and larger interface, which promotes carrier separation while also effectively inhibiting the photocorrosion of Cu_2_O. Correspondingly, more electrons are retained on the CB of ZCS, with part of them fully driving the proton reduction to produce hydrogen and the other part being transferred to Ni(OH)_2_ for the reduction reaction. The 3D hollow structure provides close contact between semiconductors and the large interfacial area, as well as creating dual channels for charge transfer and shortened diffusion distances, which are important factors for promoting a high photocatalytic activity. Based on the above experimental results, the p‐n junction between ZCS and Cu_2_O─S facilitates the transfer of photogenerated electrons from Cu_2_O─S to the ZCS conduction band. The electrons are then transferred to Ni(OH)_2_ for the reduction of water protons to generate H_2_. GO effectively assists the rapid migration of photogenerated electrons between Cu_2_O─S, ZCS, and Ni(OH)_2_. Therefore, the direct and rapid migration of photogenerated electrons in the Cu_2_O─S@GO@Zn_0.67_Cd_0.33_S─Ni(OH)_2_ photocatalyst significantly enhances the photocatalytic H_2_ evolution performance.

## 3 Conclusion

In summary, we successfully synthesized a Cu_2_O─S@GO@Zn_0.67_Cd_0.33_S─Ni(OH)_2_ 3D hollow heterojunction with unique electron‐directed transfer. After optimizing the contents of Cu_2_O─S and Ni, the ideal composite (7% CSGZCS‐4.5N) achieved a visible‐light‐driven H_2_ production rate of 48.5 mmol g^−1^ h^−1^ and an apparent quantum efficiency of 36.2% under irradiation at 420 nm by these 3D heterostructures, 97 times higher than that obtained from traditional Zn_0.67_Cd_0.33_S nanostructure. Furthermore, the H_2_ production rate reached 77.3 mmol g^−1^ h^−1^ without cooling, verifying the effectiveness of the photothermal effect. A localized built‐in electric field was formed at the interface of Cu_2_O─S and Zn_0.67_Cd_0.33_S, which partially induced the generation of strong electromagnetic fields and high‐energy charges around them and promoted the spatial separation of charges. In addition, the surface photodeposition of Ni^2+^ provided abundant active sites that promoted further rapid flow of electrons to the surface reaction, achieving precise modulation of photogenerated charges. Significantly, the heterojunction construction and the surface loading of nonprecious metals synergistically enhanced the photothermal effect. This work provides a new research avenue for the rational design and development of novel photocatalysts for efficient solar energy conversion.

## 4 Experimental Section

### Chemicals

All reagents (analytical grade) were purchased directly from Aladdin Industrial Corporation and used without further purification. Deionized water was used in the experiments.

### Synthesis of Cu_2_O Dodecahedral Structures

In a typical synthesis, a CuCl_2_ aqueous solution (0.01 mol L^−1^, 100 mL) was heated to 55 °C in a water bath, and 10 mL NaOH aqueous solution (2 mol L^−1^) was added dropwise. The solution immediately turned blue‐green and then dark brown. After constant stirring for 0.5 h, an ascorbic acid aqueous solution (0.6 mol L^−1^, 10 mL) was added dropwise to form a red turbid solution. After continued stirring for 3 h, a reddish‐brown precipitate was obtained. The precipitate was washed with distilled water and ethanol. Finally, the obtained product was dried at 60 °C under vacuum conditions for 5 h to obtain Cu_2_O polyhedra with special (100)‐truncated rhombic dodecahedral morphology.

### Synthesis of Cu_2_O─S Truncated Rhombic Dodecahedra

Cu_2_O─S truncated rhombic dodecahedra were prepared using the anion exchange method.^[^ ^37^ ^]^ Cu_2_O powder (15 mg) was dispersed in 25 mL of water and sonicated for 0.5 h to form a homogeneous solution. The sample was then stirred in an ice water bath at 5 °C for 5 min, and injected with 180 µL and 1200 µL of Na_2_S·9H_2_O aqueous solution (0.1 mol L^−1^) for another 15 min, respectively. The precipitate was then collected by high‐speed centrifugation and washed with water and ethanol. Finally, the catalysts were dried under vacuum for 10 h, and the resulting products were named Cu_2_O─S and Cu_2_O─S/2, respectively.

### Synthesis of Cu_2_O─S@GO@Zn_0.67_Cd_0.33_S 3D heterojunctions

First, a Cu_2_O─S@GO suspension was prepared by ultrasonication. The Cu_2_O─S powder was uniformly dispersed into an aqueous solution by sonication for 2 h. A GO solution (1 mg L^−1^) was added dropwise, and a Cu_2_O─S@GO suspension solution was formed by continuous sonication for 1 h. Then, the Cu_2_O─S@GO@Zn_0.67_Cd_0.33_S composites were prepared by hydrothermal method. Six millimoles of L‐cysteine were added to 100 mL of H_2_O and stirred for 10 min. One millimole of Zn(NO_3_)_2_·6H_2_O and 0.5 mmol of Cd(NO_3_)_2_·4H_2_O were added sequentially into the above solution and stirred vigorously for 30 min. The solution was separated into two parts of 50 mL and diluted to 60 mL, and a certain amount of Cu_2_O─S@GO suspension was added to one part and stirred for 30 min. The solution was transferred to a 100 mL autoclave and heated in an oven at 130 °C for 6 h. After the reaction was completed, the solution was cooled to room temperature. The sample was washed with water and ethanol, followed by drying under vacuum at 60 °C for 12 h. The obtained powder was named x% CSGZCS (x = 3, 5, 7, 9, and 11, where x% denotes the mass ratio of Cu_2_O─S@GO to Zn_0.67_Cd_0.33_S).

The pure sample, named Zn_0.67_Cd_0.33_S (ZCS), was prepared without Cu_2_O─S@GO suspension in the above solution. The Cu_2_O@GO suspension was obtained by sonication of Cu_2_O with GO under the same conditions listed above, and the sample subsequently composited with ZCS was named x% COGZCS (x = 7, where x% denotes the mass ratio of Cu_2_O@GO to Zn_0.67_Cd_0.33_S). In addition, the Cu_2_O─S suspension directly prepared without the addition of GO was compounded with ZCS, and the obtained sample was labeled x% CSZCS (x = 7, where x% denotes the mass ratio of Cu_2_O─S to Zn_0.67_Cd_0.33_S).

### Synthesis of Cu_2_O─S@GO@Zn_0.67_Cd_0.33_S─Ni(OH)_2_ Nanohybrids

Cu_2_O─S@GO@Zn_0.67_Cd_0.33_S─Ni(OH)_2_ nanohybrids were obtained via an in situ photodeposition method. First, 20 mg of 7% Cu_2_O─S@GO@Zn_0.67_Cd_0.33_S powder was dispersed into a 100 mL aqueous solution containing 0.35 m Na_2_S/0.25 m Na_2_SO_3_ and ultrasonically dispersed. Then, a certain amount of Ni(CH_3_COO)_2_·4H_2_O (3 mg mL^−1^) solution was added dropwise to the above suspension under stirring. Finally, the photodeposition reaction was carried out during a photocatalytic hydrogen evolution process. The resulting Cu_2_O─S@GO@Zn_0.67_Cd_0.33_S‐y wt.% Ni(OH)_2_ was named 7% CSGZCS‐y N (y = 3, 4.5, and 6, where x wt.% denotes the mass ratio of Ni^2+^ to Cu_2_O─S@GO@Zn_0.67_Cd_0.33_S). And the obtained sample was named CSGZCS‐N.

### Photocatalytic H_2_ Production

Photocatalytic hydrogen production tests were performed in a specific hydrogen production apparatus (CELSPH2N, Beijing China Education Au‐light Co., Ltd.). The catalyst powder (20 mg) was weighed exactly and added to 100 mL of 0.25 m Na_2_SO_3_ and 0.35 m Na_2_S·9H_2_O aqueous solution; then, the mixed solution was ultrasonically dispersed and transferred to a quartz reactor (250 mL). The reactor was then connected to the hydrogen production unit, equipped with a cooling water circulator (LX‐300, NO: N201208A1964) to provide a constant temperature of 6 °C, 10 °C, 15 °C and 25 °C,respectively. A 300 W xenon lamp with a 420 nm cutoff filter was used as a light source during the reaction. The hydrogen yields were evaluated by gas chromatography (GC‐7920, TCD, Ar carrier gas), using the related equations.

In addition, for the calculation of the apparent quantum efficiency (AQE), the amount of hydrogen produced was measured with a monochromatic light filter using a series of single‐wavelength filters (360, 420, and 450 nm) under the same test conditions. The AQE of the catalysts was calculated as:^[^ ^11^ ^]^

(1) $$\mathrm{AQE}\;=\frac{2\times \;\mathrm{the}\;\mathrm{number}\;\mathrm{of}\;\mathrm{evolved}\;H_{2}\;\mathrm{molecules}}{\mathrm{the}\;\mathrm{number}\;\mathrm{of}\;\mathrm{incident}\;\mathrm{photons}}\;\times 100\%$$

### Characterizations

The crystalline structure of the samples was determined by X‐ray diffraction (Empyrean, Panalytical, Holland). Fine measurements (40–48° 1°/min) were made using Double crystal XRD. The Raman spectra were investigated at the excitation wavelength of 325 nm obtained from a He–Ne laser on a LABRAM HR EVO Micro‐Raman spectrometer. The apparent morphology of the samples was obtained using a Scanning Electron Microscopy (SUPRA55, Carl Zeiss, Germany). The TEM images were taken on a Transmission Electron Microscopy (FEI Talos F200XG2 AEMC, Thermo Fisher Scientific, USA). The UV–vis diffuse reflectance absorption spectra (UV–vis DRS, the diffuse reflectance measurement model QE‐C2) and the fluorescence spectra (PL, an excitation wavelength of 266 nm) of the samples were measured using the Solar Quantum Efficiency Test System (SCS10‐X150‐DSSC, Zolix Instruments Co., Ltd.). To analyze the surface chemical valence of the elements in the catalyst, the samples were determined by an X‐ray photoelectron spectrometer (Thermo Scientific K‐Alpha, Thermo Fisher Scientific, USA). Among them, Al‐Kα was used as the X‐ray source, and C1s (284.8 eV) energy spectrum was used for energy correction. The time‐resolved photoluminescence (TRPL) spectra were tested by a transient‐steady‐state fluorescence spectrometer (Edinburgh FLS1000, UK) with an excitation wavelength of 405 nm. The content of an element in the catalyst was obtained using an Inductively coupled plasma mass spectrometry (ICP‐MS, Agilent 7700, USA). Infrared thermographic testing of the catalysts was performed on a Thermal imaging camera (FLIR T540, USA). The ultraviolet photoelectron spectroscopy (UPS) spectra of the samples were tested on a ThermoFisher ESCALAB250Xi (external 10 eV bias). Brunauer‐Emmett‐Taylor surface area (S_BET_) and pore size distribution were performed on the Fully Automated Specific Surface Area Analyzer (ASAP2460, USA). In situ, the XPS energy spectrum was collected using the X‐ray photoelectron spectrometer (PHI5000 VersaprobeIII XPS, Japan). The X‐ray source was a Mono AlKa source and an energy of 1486.6 eV. Zeta potential was conducted using the Nanoparticle Size and Zeta Potential Analyzer (Malvern Zetasizer Nano ZS90, UK).

### Photoelectrochemical Measurements

The photocurrent response test and impedance test of the photocatalyst were obtained on an electrochemical workstation (CHI660E, Chenhua, China) by using a conventional three‐electrode system. First, the working electrode was prepared by the spin‐coating method. The homogeneous solution of the sample was uniformly applied to the conductive surface of the conductive glass (ITO, 10*15 mm). Then the prepared working electrode was calcined at 100 °C for 2 h under but nitrogen atmosphere to make a closer contact between the sample and the conductive surface. Finally, the prepared electrode, platinum sheet electrode, and Ag/AgCl electrode corresponded to the working electrode, counter electrode, and reference electrode in the three‐electrode system, respectively. Na_2_SO_4_ (0.1 mol L^−1^) solution was chosen as the electrolyte. A 300 W xenon lamp (PLS‐SXE300, Beijing trusttech Co., Ltd.) with a cut‐off filter (λ ≥ 420 nm) was used as the excitation light source.

The conversion formula for the different electrode potentials is as follows:^[^ ^22^ ^]^

(2) $$E\;\left(\mathrm{versus}\;\mathrm{NHE}\right)=\;E\;\left(\mathrm{vs}\;\mathrm{Ag}/\mathrm{AgCl}\right)+\;0.197$$

### Theoretical Calculations

The spin‐polarized DFT calculations were carried out with the Vienna ab initio simulation package (VASP).^[^ ^60^ ^]^ The ion‐electron interactions were described by the projector augmented wave (PAW) method.^[^ ^61^ ^]^ The Perdew–Burke–Ernzerhof (PBE) functional within a generalized gradient approximation (GGA) was used to describe the electron exchange‐correlation interaction.^[^ ^62^ ^]^ A kinetic cutoff energy of 450 eV was adopted. The convergence thresholds of the energy and residual force for the structural optimizations were set as 10^−5^ eV and 0.02 eV Å^−1^, respectively. The empirical corrective Grimme's scheme (DFT‐D3) method was used to treat the van der Waals interactions.^[^ ^63^ ^]^ The *k*‐point of the first Brillouin zone was sampled with a Monkhorst‐Pack^[^ ^64^ ^]^ mesh of 3 × 3 × 1 for structural optimizations. Bader charge analysis was employed to measure the charge transfer.^[^ ^65^ ^]^ The slab models of Cu_2_O(100) and Zn_0.67_Cd_0.33_S(101) surfaces were used to build the Cu_2_O/Zn_0.67_Cd_0.33_S heterojunction (Figure S15, Supporting Information), where a vacuum thickness larger than 15 Å was set along the *z*‐axis to avoid the interaction between periodic images. An interface O atom in Cu_2_O/Zn_0.67_Cd_0.33_S was substituted by an S atom to simulate the Cu_2_O─S/Zn_0.67_Cd_0.33_S heterojunction model, where the most stable configuration was selected (Figure S21, Supporting Information).

The absorption free energy of hydrogen atom (Δ*G*_H*_) was calculated based on the theory proposed by Nørskov et al.^[^ ^66^ ^]^ with the following formula:

(3) $$\Delta G_{H*}=\;\Delta E_{H*}\;+\Delta E_{\mathrm{ZPE}}-T\Delta S$$

where, Δ*E*_H*_ is the reaction energy change directly obtained from DFT calculations. Δ*E*_ZPE_ and Δ*S* are the changes in zero‐point energy and entropy, respectively, which could be acquired from the NIST database, and the relevant values are listed in Table S1 (Supporting Information). The temperature *T* was set to 298.15 K.

## Conflict of Interest

The authors declare no conflict of interest.

## Supporting information

Supporting Information

## Data Availability

The data that support the findings of this study are available from the corresponding author upon reasonable request.
