# Supplementary material for: Rational Design of Three Dimensional Hollow Heterojunctions for Efficient Photocatalytic Hydrogen Evolution Applications
Source: Adv Sci (Weinh). 2024 Jan 23;11(13):2309293. doi: 10.1002/advs.202309293 (PMC10987164; doi:10.1002/advs.202309293)
Supplement: Supplementary file 1 — Supporting Information [file ADVS-11-2309293-s001.pdf]

ADVANCED SCIENCE Open Access

Supporting Information

for *Adv. Sci.*, DOI 10.1002/advs.202309293

Rational Design of Three Dimensional Hollow Heterojunctions for Efficient Photocatalytic Hydrogen Evolution Applications

Jingwen Pan, Dongbo Wang, Donghai Wu*, Jiamu Cao*, Xuan Fang*, Chenchen Zhao, Zhi Zeng, Bingke Zhang, Donghao Liu, Sihang Liu, Gang Liu*, Shujie Jiao, Zhikun Xu*, Liancheng Zhao* and Jinzhong Wang**

Supporting Information

Rational Design of Three-Dimensional Hollow Heterojunctions for Efficient Photocatalytic Hydrogen Evolution Applications

Jingwen Pan, Dongbo Wang, Donghai Wu*, Jiamu Cao*, xuan Fang*, Chenchen Zhao, Zhi Zeng, Bingke Zhang, Donghao Liu, Sihang Liu, Gang Liu*, Shujie Jiao, Zhikun Xu*, Liancheng Zhao*, Jinzhong Wang**

Table S1 The relevant values of ΔE_{ZPE} and ΔS

H_2	
0.27	ZPE
-0.41	TS
-6.766	E
-6.906	G

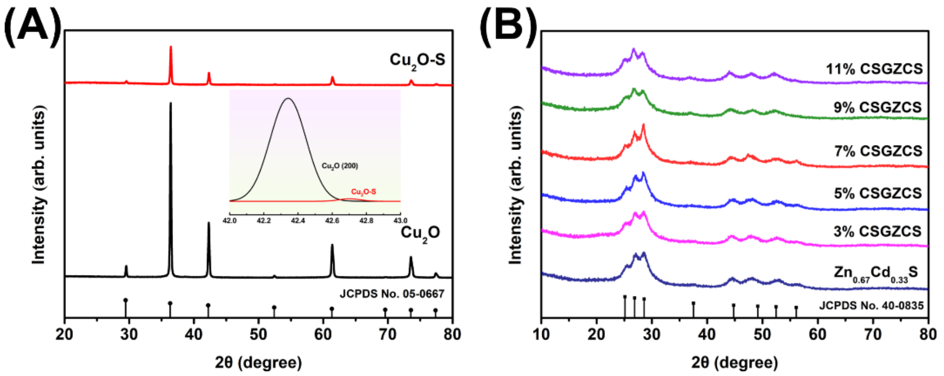

Figure S1. A) XRD patterns of Cu_2O, Cu_2O-S (The inset shows Double crystal XRD of Cu_2O and Cu_2O-S) and B) XRD patterns of $Zn_{0.67}Cd_{0.33}S$ and x% CSGZCS composite (x=3, 5, 7, 9 and 11).

Since Cu_2O (200) 2θ locate at 42.2°, Cu_2S (200) 2θ locate at 46.3°.[S1, S2]

The lattice constants of $Cu_2O_xS_{1-x}$ are estimated:

$$42.2^{o}\times X+46.3^{o}\times(1-X) = 42.56^{o}$$

Therefore, the diffraction peak at 42.6° is belong to $Cu_2O_xS_{1-x}$.

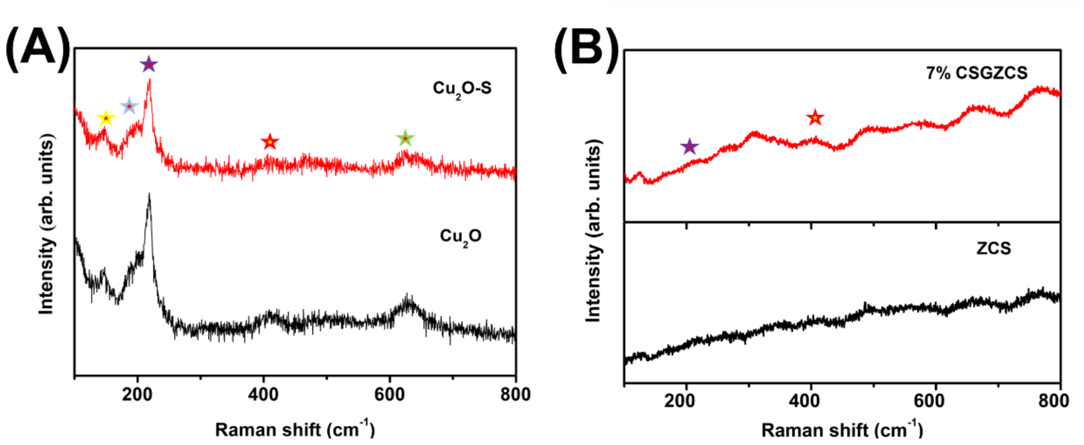

Figure S2. Raman spectrum of A) Cu_2O, Cu_2O-S and B) $Zn_{0.67}Cd_{0.33}S$ and 7% CSGZCS.

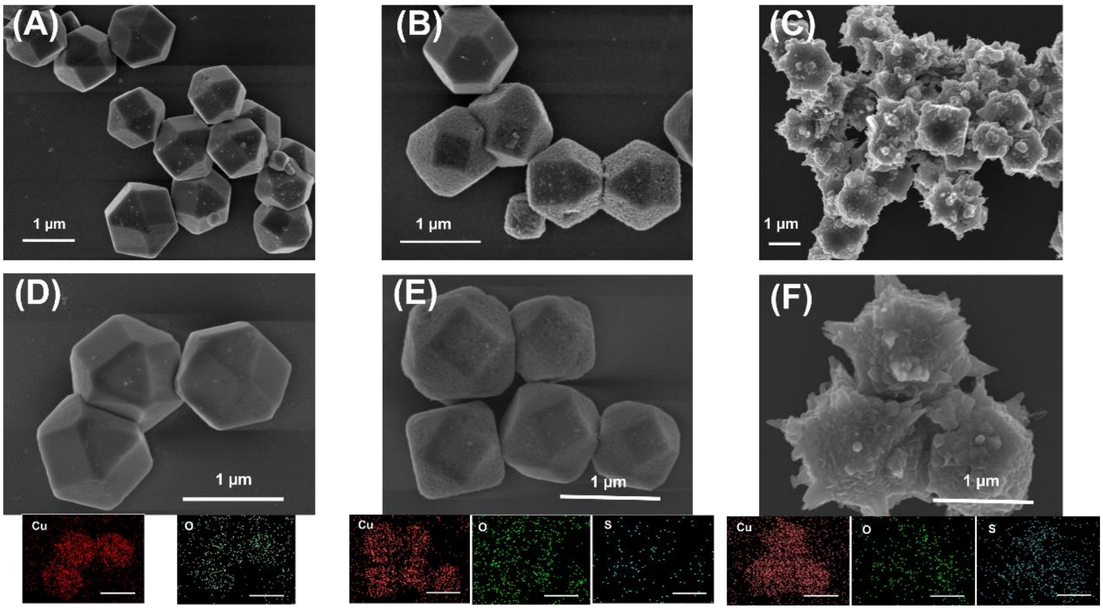

Figure S3. SEM image and corresponding mapping image of A, D) Cu_2O, B, E) Cu_2O-S and C, F) Cu_2O-S/2.

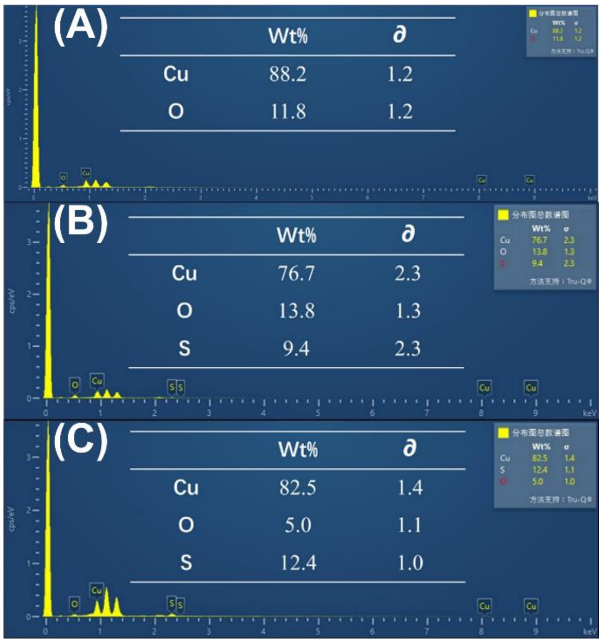

Figure S4. EDX spectrum of A) Cu_2O, B) Cu_2O-S and C) Cu_2O-S/2 from SEM pictures in figure S3 (the illustrated table shows the corresponding elemental content).

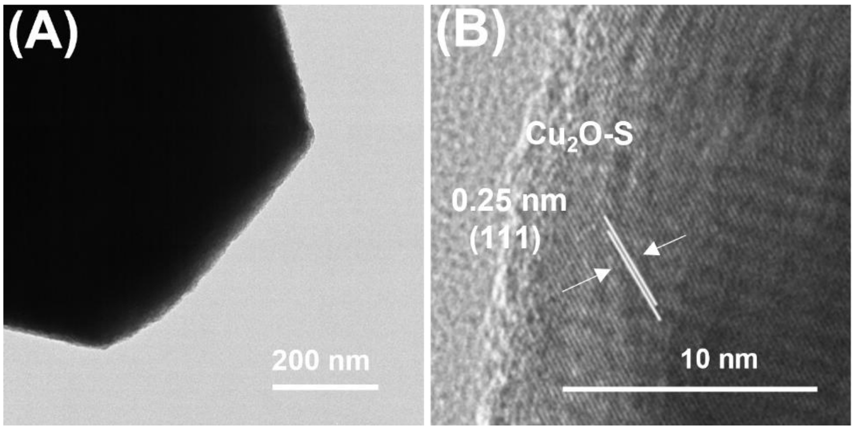

Figure S5. HRTEM image of Cu_2O-S.

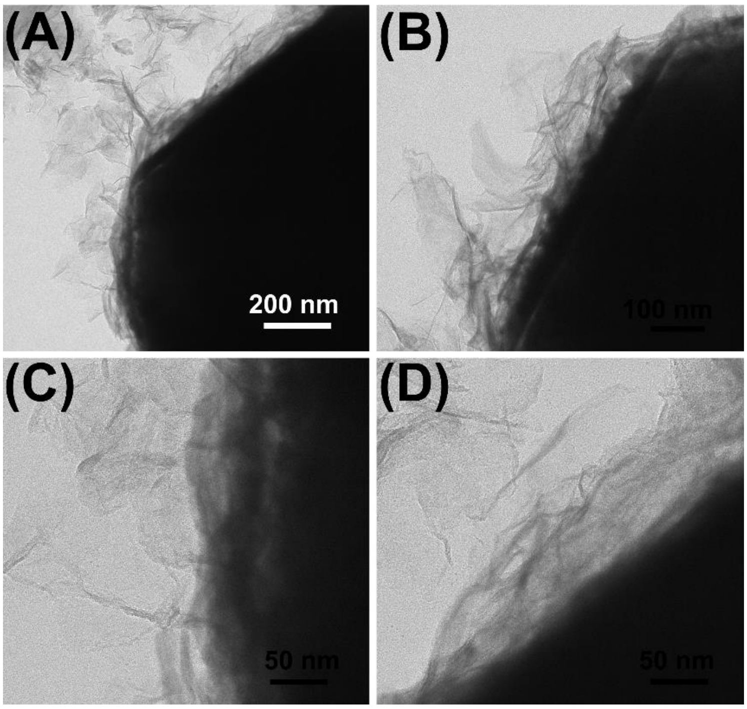

Figure S6. TEM image of Cu_2O-S@GO.

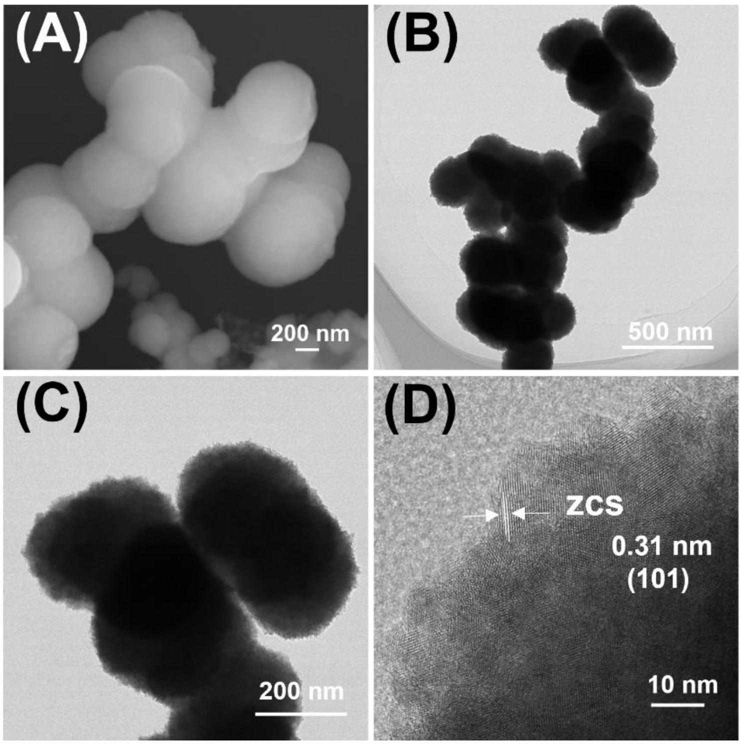

Figure S7. A) SEM, B, C) TEM and D) HRTEM of $Zn_{0.67}Cd_{0.33}S$.

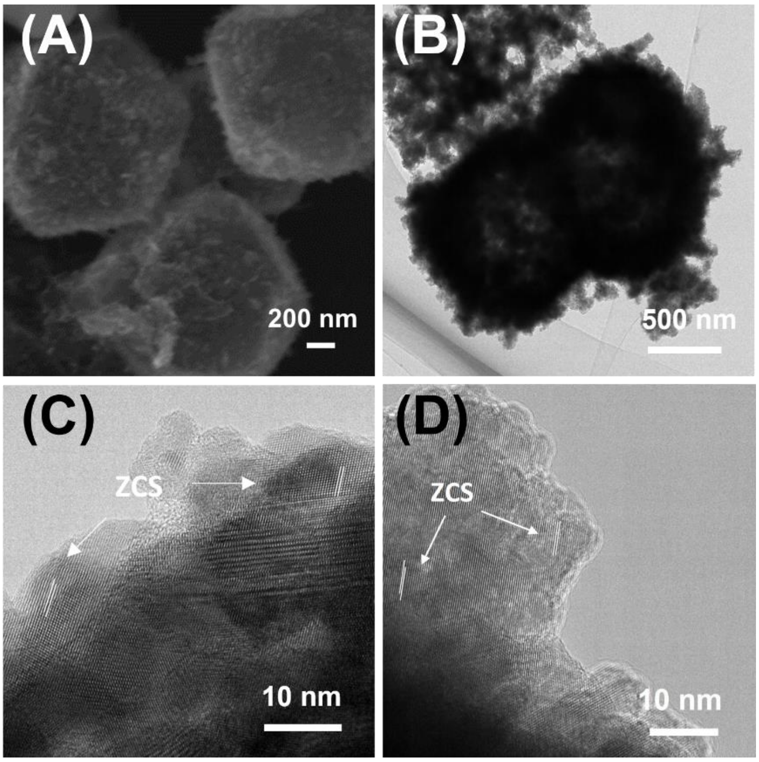

Figure S8. A) SEM, B, C) TEM and D) HRTEM of 7% CSGZCS.

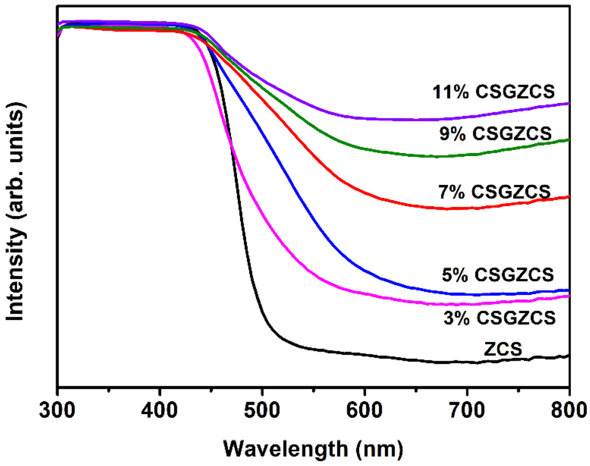

Figure S9. UV-vis diffuse reflectance spectra of ZCS and x% CSGZCS (x=3, 5, 7, 9 and 11).

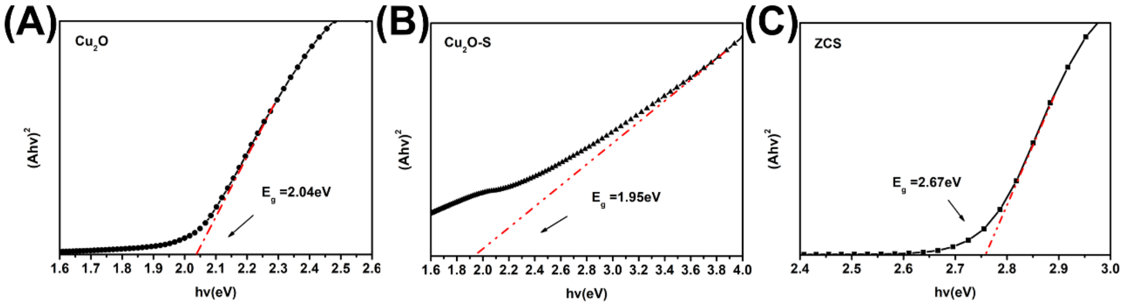

Figure S10. The calculated bandgap of A) Cu_2O, B) Cu_2O-S and C) ZCS.

Table S2 Kinetic parameters of PL decay for ZCS, CSZCS, COGZCS and CSGZCS

Samples	τ_1 (ns)	B_1	τ_2 (ns)	B_2	τ_3 (ns)	B_3	τ (ns)	χ^2
ZCS	0.49	2202.76	2.32	1132.68	8.6	69.89	2.8	1.15
CSZCS	0.88	2544.67	5.51	219.12	83.99	2.82	33.6	1.14
COGZCS	0.89	2706.03	8.89	324.71	96.05	27.37	35.2	1.12
CSGZCS	0.84	2841.46	7.09	154.32	73.71	55.68	41.1	1.11

The formula for calculating the average value of τ is as follows:

$$\tau = \frac{\tau_1^2 \times B_1 + \tau_2^2 \times B_2 + \tau_3^2 \times B_3}{\tau_1 \times B_1 + \tau_2 \times B_2 + \tau_3 \times B_3}$$

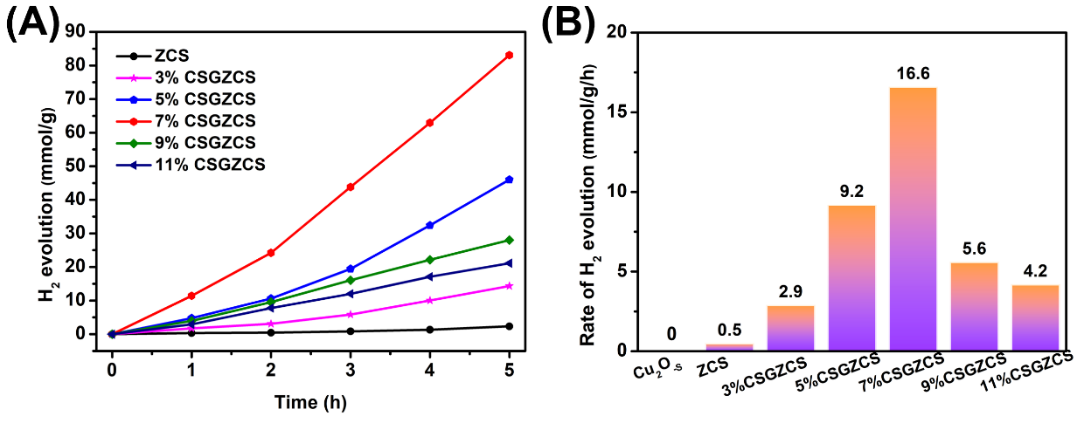

Figure S11. A) Photocatalytic H_2 evolution activities and B) corresponding average rates of ZCS and x% CSGZCS (x=3, 5, 7, 9 and 11).

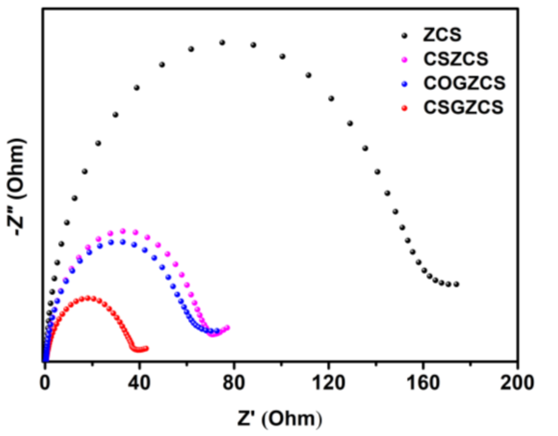

Figure S12. EIS spectra of the ZCS, CSZCS, COGZCS, and CSGZCS samples.

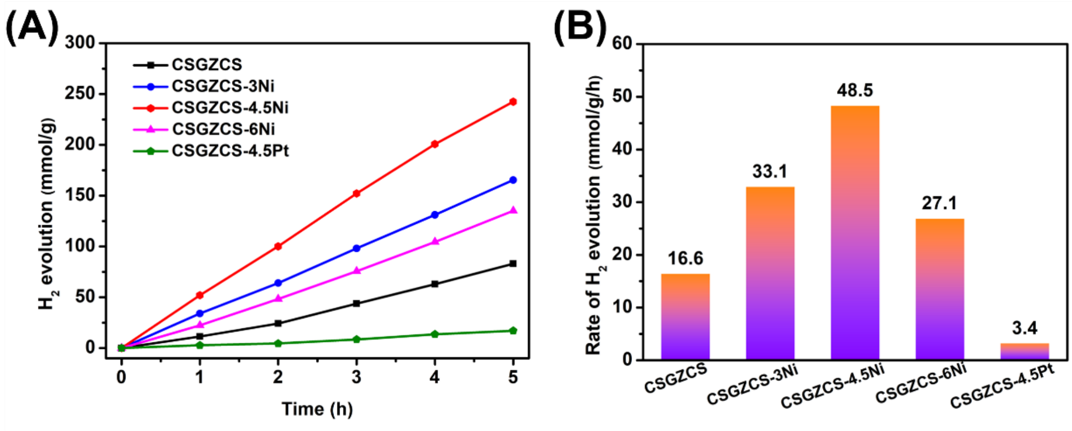

Figure S13. A) Photocatalytic H_2 evolution activities and B) corresponding average rates of CSGZCS, x wt% Ni^{2+} loaded 7% CSGZCS (x=3, 4.5, 6) and 4.5 wt.% Pt loaded 7% CSGZCS (7% CSGZCS-4.5Pt).

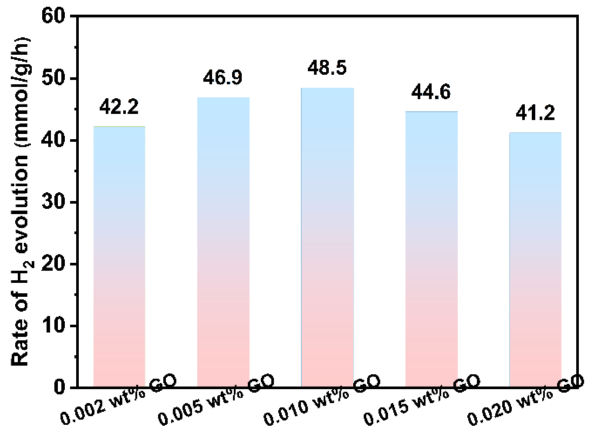

Figure S14. Comparison of photocatalytic H_2 evolution activities of Cu_2O-S@GO@ZCS-$Ni(OH)_2$ composites with different theoretical contents of GO.

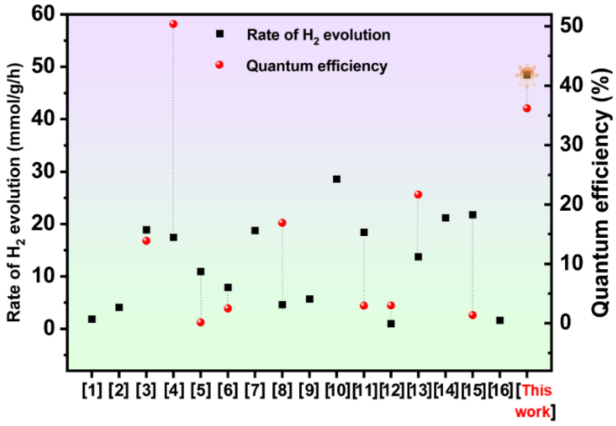

Figure S15. A brief summary of relevant types of catalysts in recent years.

Table S3 A brief summary table of the relevant types of catalysts mentioned above in recent years.

Photocatalysts	Hole scavenger	Light source	Maximum rate (mmol·h^{-1}·g^{-1})	AQE%	Ref.
N-TiO_{2-x}@MoS_2 (3D)	CH_3OH	AM1.5	1.882	-	S3
TiO_2/g-C_3N_4-Pt (3D)	TEOA	350W Xe lamp	4.128	-	S4
NiS/CdS	Lactic acid	300W Xe lamp λ> 420 nm	18.9 (7°C)	13.9 (420 nm)	S5
$Pt_{0.3}$-$ZnIn_2S_4$	TEOA	300W Xe lamp λ> 420 nm	17.5 (8°C)	50.4 (420 nm)	S6
NiIn-$ZnIn_2S_4$	TEOA	300W Xe lamp λ> 420 nm	10.97	0.148 (400 nm)	S7
CuS@$ZnIn_2S_4$ (3D)	Na_2S/Na_2SO_3	300W Xe lamp λ> 400 nm	7.91 (25°C)	2.52 (420 nm)	S8
UiO-66 $(COOH)_2$/MoS_2/$ZnIn_2S_4$ (3D)	Lactic acid	300W Xe lamp -	18.794 (25°C)	-	S9
$Cu_{2-x}S$@$ZnIn_2S_4$ (3D)	Na_2S/Na_2SO_3	300W Xe lamp -	4.653	16.9 (420 nm)	S10
$Zn_{0.6}Cd_{0.4}S$ hollow cage (3D)	Lactic acid	300W Xe lamp λ> 420 nm	5.68	-	S11
$Zn_{0.5}Cd_{0.5}S$/ZnO/$Zn_{0.5}Cd_{0.5}S$ (3D)	Na_2S/Na_2SO_3	300W Xe lamp	28.6 (25°C)	-	S12
CoV-LDH@ $Zn_xCd_{1-x}S$ (3D)	Na_2S/Na_2SO_3	5 W LED -	18.438	2.96 (420 nm)	S13
NiFe-LDH/$Zn_{0.5}Cd_{0.5}S$ (3D)	Lactic acid	300W Xe lamp λ> 420 nm	1.017	3.0 (420 nm)	S14

ZnCdS/Co-MoS_x	Lactic acid	300W Xe lamp λ> 420 nm	13.787 (5°C)	21.7 (420 nm)	S15
ZnCdS/Pt-CrO_x	TEOA	300W Xe lamp λ> 420 nm	21.2 (10°C)	-	S16
Mo_2C/Au@$Zn_{0.5}Cd_{0.5}S$	Lactic acid	300W Xe lamp λ> 420 nm	21.819 (4°C)	1.39 (420 nm)	S17
Cu_2O/Cu_7S_4	Na_2SO_3	300W Xe lamp -	1.689	-	S18
Cu_2O-S@GO@$Zn_{0.67}Cd_{0.33}S$-$Ni(OH)_2$	Na_2S/Na_2SO_3	300W Xe lamp λ>420 nm	48.5 (10°C)	36.2	This work

(The temperature is the external condensation temperature for photocatalytic hydrogen production)

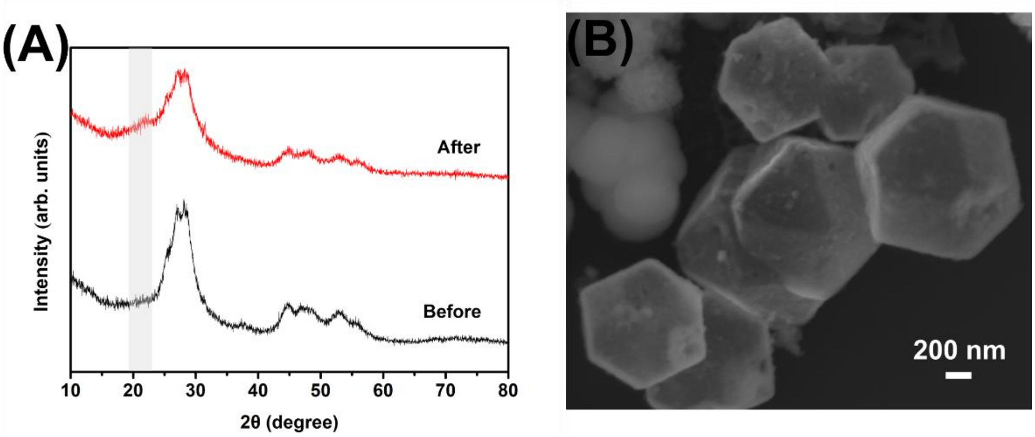

Figure S16. A) XRD patterns of 7%CSGZCS before and after cycle reaction, B) SEM of 7%CSGZCS after cycle reaction.

ZCS.mp4

CSGZCS.mp4

CSZCS-N.mp4

CSGZCS-N.mp4

Figure S17. Infrared thermal imaging video of ZCS, CSGZCS, CSZCS-N and CSGZCS-N after 30 min.

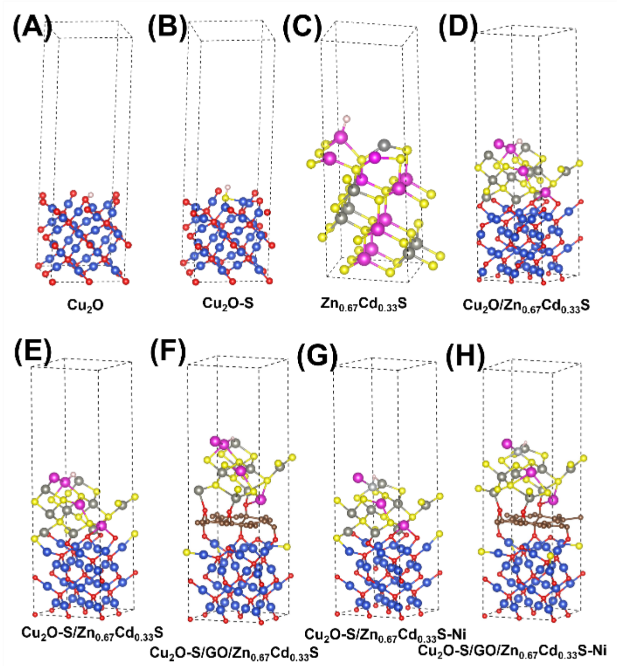

Figure S18. The corresponding H adsorption configurations of Cu_2O, Cu_2O-S, $Zn_{0.67}Cd_{0.33}S$, $Cu_2O/Zn_{0.67}Cd_{0.33}S$, Cu_2O-S/$Zn_{0.67}Cd_{0.33}S$, Cu_2O-S/GO/$Zn_{0.67}Cd_{0.33}S$, Cu_2O-S/$Zn_{0.67}Cd_{0.33}S$-Ni and Cu_2O-S/GO/$Zn_{0.67}Cd_{0.33}S$-Ni heterojunction.

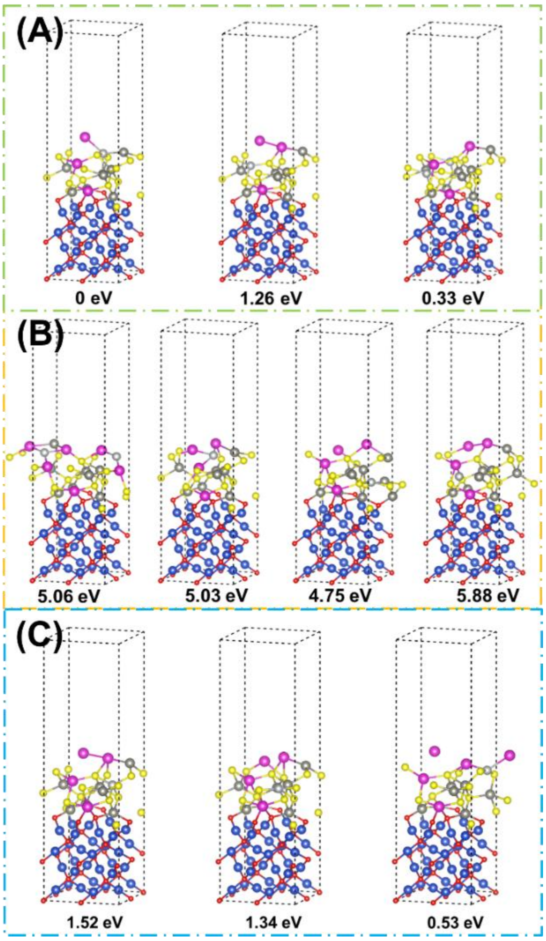

Figure S19. The most stable Ni doping sites on the surface of the Cu_2O-S/$Zn_{0.67}Cd_{0.33}S$

heterojunction based on A) Ni substituted Cd, B) Ni substituted S and C) Ni substituted Zn sites.

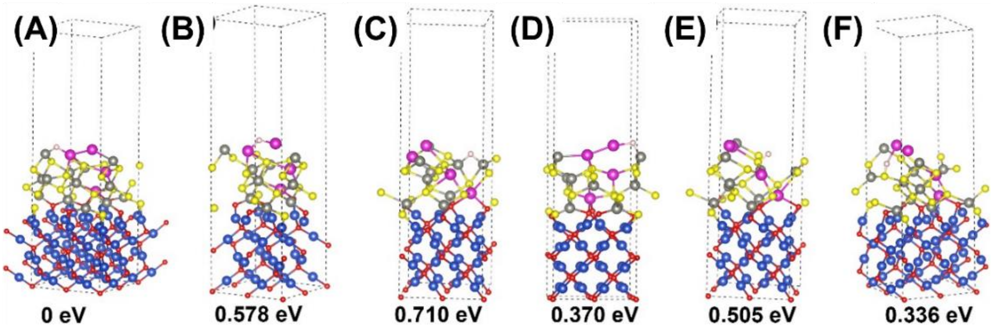

Figure S20. The optimized configurations of H atoms adsorption on the surfaces of Cu_2O-S/$Zn_{0.67}Cd_{0.33}S$ heterojunction. The black values are the total energies with respect to figure A.

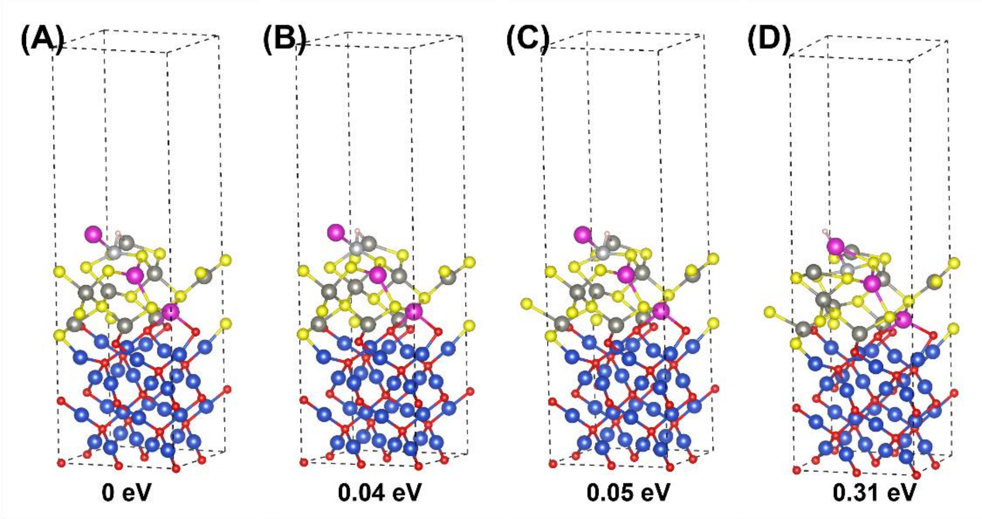

Figure S21. The optimized configurations of H atoms adsorption on the surfaces of Cu_2O-S/$Zn_{0.67}Cd_{0.33}S$-Ni heterojunction. The black values are the total energies with respect to Figure A.

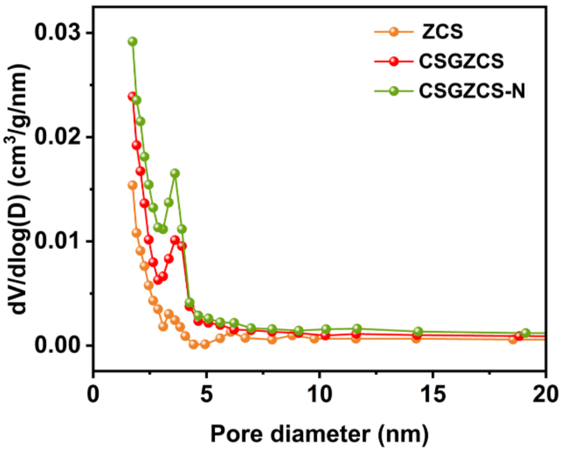

Figure S22. Pore size distribution curves of different photocatalysts.

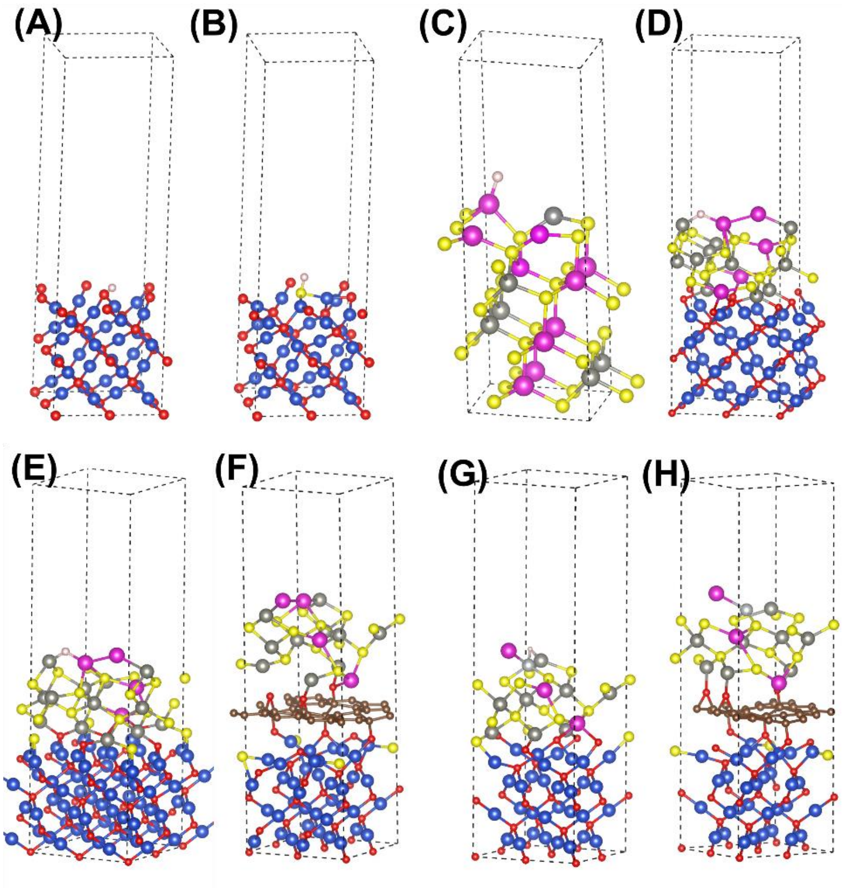

Figure S23. The optimized slab models of A) Cu_2O (100) surface, B) Cu_2O-S (100) surface, C) $Zn_{0.67}Cd_{0.33}S$ (101) surface, D) $Cu_2O/Zn_{0.67}Cd_{0.33}S$ heterojunction, E) Cu_2O-S/$Zn_{0.67}Cd_{0.33}S$ heterojunction, F) Cu_2O-S/GO/$Zn_{0.67}Cd_{0.33}S$, G) Cu_2O-S/$Zn_{0.67}Cd_{0.33}S$-Ni, and H) Cu_2O-S/GO/$Zn_{0.67}Cd_{0.33}S$-Ni heterojunction.

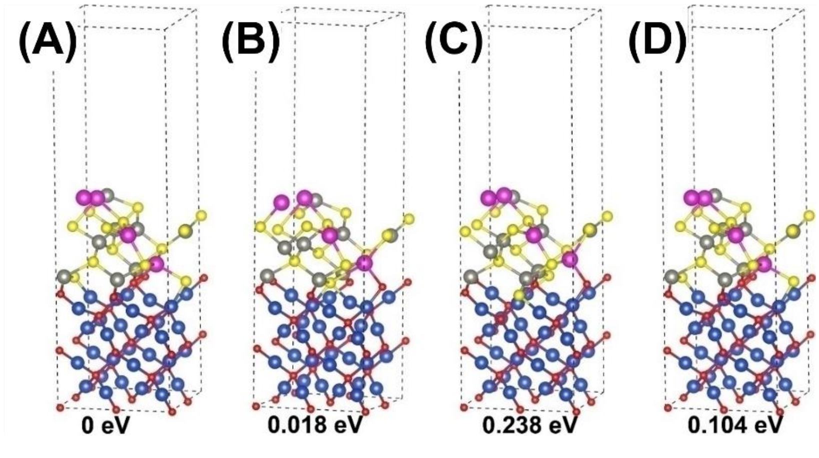

Figure S24. The optimizied configurations of S doped on the interface of $Cu_2O/Zn_{0.67}Cd_{0.33}S$ heterojunction. The black values are the total energies with respect to figure A.

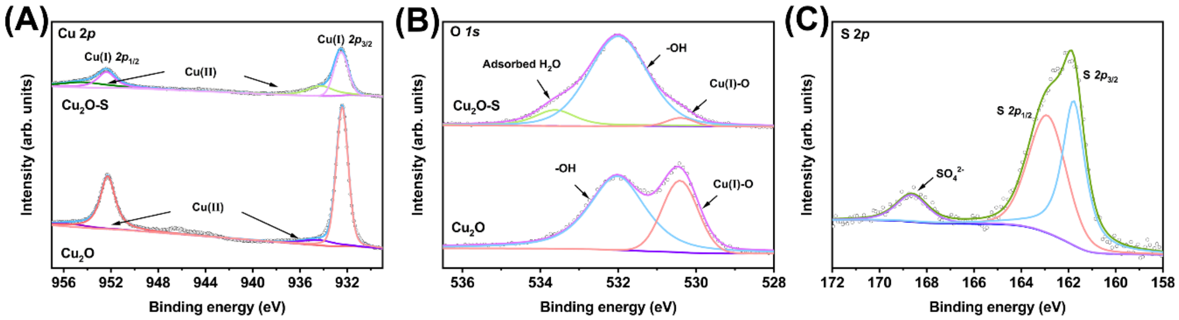

Figure S25. A) Cu 2p, B) O 1s spectra of Cu_2O, Cu_2O-S and C) S 2p spectra of Cu_2O-S.

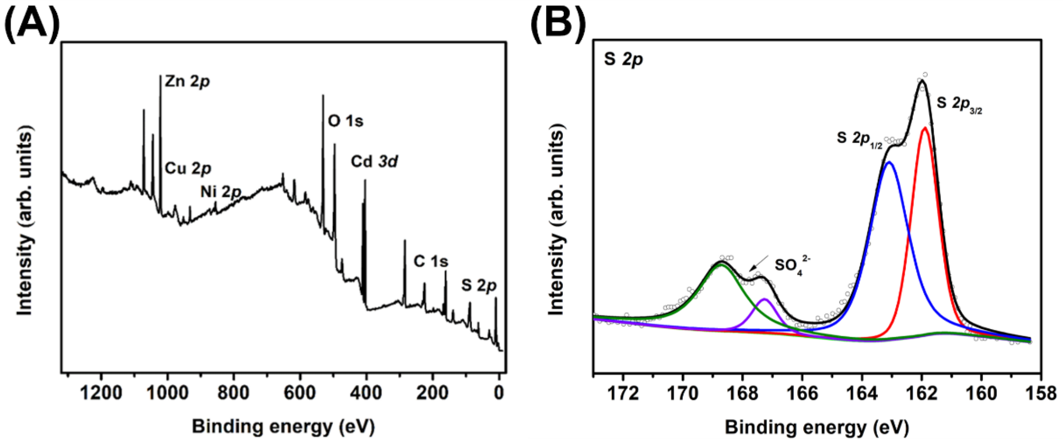

Figure S26. A) XPS spectra, and B) S 2p XPS spectrum of 7% CSGZCS-4.5N.

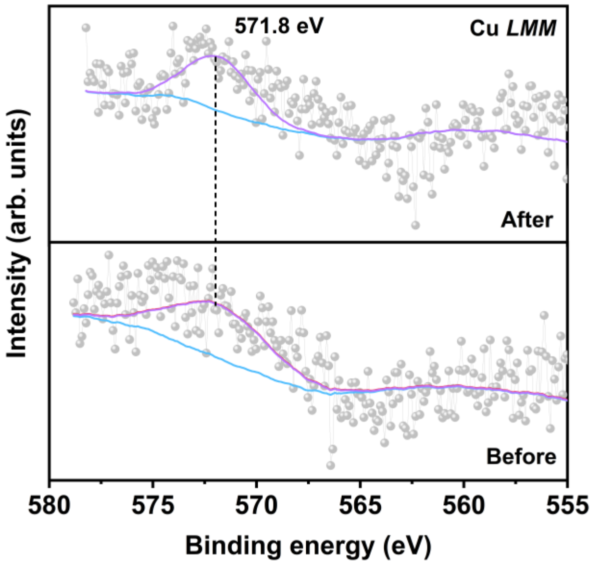

Figure S27. AES for Cu LMM of the 7% CSGZCS-4.5Ni before and after the reaction.

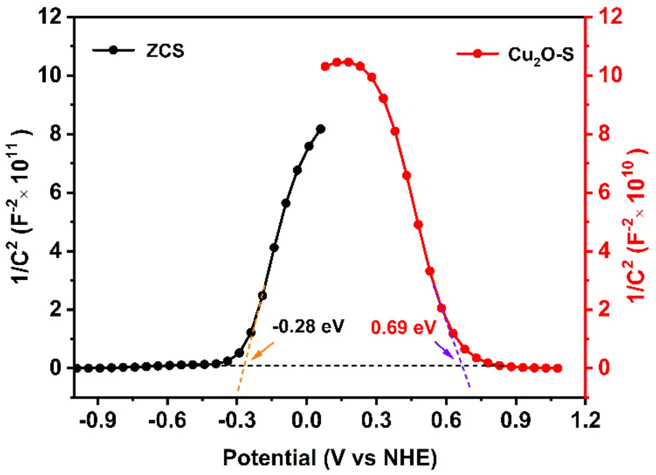

Figure S28. Mott–Schottky plots of ZCS and Cu_2O-S.

References

[S1] M. Zakeri, M.R. Rahimipour, *Adv. Powder Technol.* **2012**, *23*, 298–304.

[S2] Z. Li, Z. Zhang, Nano Res. **2018**, *11*, 1530–1540.

[S3] X. F. Liu, Z. P. Xinga, Y. Zhang, Z. Z. Li, X. Y. Wu, S. Y. Tan, X. J. Yu, Q. Zhu, W. Zhou, *Appl. Catal. B Environ.* **2017**, *201*, 119.

[S4] J. Wang, G. H. Wang, X. Wang, Y. Wu, Y. R. Su, H. Tang, *Carbon* **2019**, *149*, 618.

[S5] K. Li, H. Pan, F. Wang, Z. G. Zhang, S. X. Min, *Appl. Catal. B Environ.* **2023**, *321*, 122028.

[S6] X. W. Shi, C. Dai, X. Wang, J. Y. Hu, J. Y. Zhang, L. X. Zheng, L. Mao, H. J. Zheng, M. S. Zhu, *Nat. Commun.* **2022**, *13*, 1287.

[S7] D. X. Zhou, X. D. Xue, X. Wang, Q. J. Luan, A. Li, L. G. Zhang, B. Z. Li, W. J. Dong, G. Wang, C. M. Hou, Ni, *Appl. Catal. B Environ.* **2022**, *310*, 121337.

[S8] H. T. Fan, Z. Wu, K. C. Liu, Wei-Sheng Liu a, *Chem. Eng. J* **2022**, *433*, 134474.

[S9] F. H. Mu, Q. Cai, H. Hu, J. Wang, Y. Wang, S. J. Zhoua, Y. Kong, *Chem. Eng. J* **2020**, *384*, 123352.

[S10] J. M. Chen, J. Y. Chen, Y. W. Li, *J. Mater. Chem. A* **2017**, *5*, 24116.

[S11] J. M. Chen, Z. R. Shen, S. M. Lv, K. Shen, R. F. Wu, X. -F. Jiang, T. Fan, J. Y. Chen, Y. W. Li, *J. Mater. Chem. A* **2018**, *6*, 19631.

[S12] X. Guo, C. C. Chang, G. R. Wang, X. Q. Hao, Z. L. Jin, *ACS Appl. Energ. Mater.* **2022**, *5*, 5064.

[S13] Y. D. Sun, X. P. Wang, Q. Fu, C. X. Pan, *ACS Appl. Mater. Inter.* **2021**, *13*, 39331.

[S14] Y. G. Lei, Y. Z. Zhang, Z. X. Li, S. Xu, J. Y. Huang, K. H. Ng, Y. K. Lai, *Chem. Eng. J* **2021**, *425*, 131478.

[S15] M. Ahmad, X. Quan, S. Chen, H. T. Yu, Z. X. Zeng, *Appl. Catal. B Environ.* **2021**, *283*, 119601.

[S16] W. H. Feng, Y. H. Lei, X. S. Wu, J. Yuan, J. H. Chen, D. F. Xu, X. C. Zhang, S. Y. Zhang, P. Liu, L. L. Zhang, B. Weng, *J. Mater. Chem. A* **2021**, *9*, 1759.
